# Ursolic Acid Protected Lung of Rats From Damage Induced by Cigarette Smoke Extract

**DOI:** 10.3389/fphar.2019.00700

**Published:** 2019-06-20

**Authors:** Li Lin, Gang Hou, Dan Han, Jian Kang, Qiuyue Wang

**Affiliations:** Department of Respiratory and Critical Care Medicine, Institute of Respiratory Disease, The First Hospital of China Medical University, Shenyang, China

**Keywords:** ursolic acid, cigarette smoke extract, chronic obstructive pulmonary disease, airway remodeling, endoplasmic reticulum stress

## Abstract

**Background:** We found previously that ursolic acid (UA) administration could alleviate cigarette smoke-induced emphysema in rats partly through the unfolded protein response (UPR) PERK-CHOP and Nrf2 pathways, thus alleviating endoplasmic reticulum stress (ERS)-associated oxidative stress and cell apoptosis. We hypothesized that other UPR pathways may play similar roles in cigarette smoke extract (CSE)-induced emphysema. So, we sought to investigate the dynamic changes and effects of UPR and the downstream apoptotic pathways. Further, we investigated whether UA could alleviate CSE-induced emphysema and airway remodelling in rats, whether and when it exerts its effects through UPR pathways as well as Smads pathways.

**Methods:** One hundred eight Sprague Dawley (SD) rats were randomly divided into three groups: Sham group, CSE group, and UA group, and each group was further divided into three subgroups, administered CSE (vehicle) for 2, 3, or 4 weeks; each subgroup had 12 rats. We examined pathological changes, analyzed the three UPR signaling pathways and subsequent ERS, intrinsic and extrinsic apoptotic pathway indicators, as well as activation of Smad2,3 molecules in rat lungs.

**Results:** Exposure to CSE for 3 or 4 weeks could apparently induce emphysema and airway remodeling in rats, including gross and microscopic changes, alteration of mean alveolar number (MAN), mean linear intercept (MLI), and mean airway thickness in lung tissue sections. UA intervention could significantly alleviate CSE-induced emphysema and airway remodeling in rats. UA exerted its effects through ameliorating apoptosis by down regulating UPR signalling pathways and subsequent apoptosis pathways, as well as, downregulating p-Smad2 and p-Smad3 molecules.

**Conclusions:** UA attenuated CSE-induced emphysema and airway remodeling, exerting its effects partly through regulation of three UPR pathways, amelioration downstream apoptotic pathways, and alleviating activation of Smad2 and Smad3.

## Background

Chronic obstructive pulmonary disease (COPD) is a severe respiratory disease characterized by chronic respiratory syndrome and airway limitation, the pulmonary functional decline is progressive and ultimately results in respiratory failure. The two main pathologic manifestations of COPD—emphysema and airway-vessel remodeling—determine the phenotype of the disease (Wada et al., 2018).

Nearly 90% of COPD cases are caused by cigarette smoke (Martinez and Han, 2012). Cigarette smoke (CS) induces an imbalance of cell proliferation and apoptosis, which is thought to be critical in the pathogenesis of COPD, and determine disease phenotype (Haw et al., 2016; Bodas et al., 2016; Lee et al., 2017a; Lee et al., 2017b). The three classical apoptotic pathways: endoplasmic reticulum stress (ERS), intrinsic, and extrinsic apoptotic pathways all have been implicated in the pathogenesis of COPD (Wu et al., 2015; Fujita et al., 2016; Yuan et al., 2017; Lin et al., 2017).

In recent years, unfolded protein response (UPR) mediated ERS associated cell apoptosis is attracting more and more researchers’ attention on its effect in development of COPD (Kelsen, 2016). Except for ERS associated apoptosis, the three ER transmembrane UPR sensors—PERK, IRE1, and ATF6—are also participant in mediating intrinsic and extrinsic apoptotic pathways, but the mechanisms remain obscure (Okumura et al., 2017; Cano-Gonzalez et al., 2018). Under ERS, PERK “unchaperonization” and autophosphorylation, phosphorylated PERK (p-PERK) changes the balance between eIF2α/p-eIF2α in such a way that it is tipped decidedly in favor of p-eIF2α. p-eIF2α, in turn, activates CHOP (Tan et al., 2017). Except for activating ERS associated apoptotic factor Cleaved-Caspase12(4) and then Cleaved-Caspase3, CHOP can also downregulate anti-apoptotic family of Bcl-2 (BH domain) and upregulate pro-apoptotic subfamily of Bcl-2 (BH3 domain). Decreased Bcl-2 (BH domain) correlates with an induction of mitochondrial-related pathway of apoptosis (called “intrinsic”) yielding in activation of Caspase9, and finally, Caspase3 (Ding et al., 2016; Iurlaro and Muñoz-Pinedo, 2016; Okumura et al., 2017; Guo et al., 2018). CHOP could also promote cell death by activating receptor-related pathway of apoptosis (called “extrinsic”) through activation of pro-apoptotic genes, including death receptor 5(DR5), and lead to the activation of Caspase8, and finally, Caspase3 (Cano-Gonzalez et al., 2018; Chang et al., 2018). Except for its adaptive effects, IRE1 pathway has also clearly been shown to be associated with intrinsic apoptosis, because surges in IRE1 pathway lead to activation of stress kinases, including c-Jun N-terminal kinase (JNK) (Joo et al., 2016). JNK has also been known to phosphorylate and modulate the activities of several Bcl-2 family proteins (Lei and Davis, 2003). Other than that, IRE1 and ATF6 also merge to induce CHOP expression and subsequent apoptosis event (Puthalakath et al., 2007; Kim et al., 2008; He et al., 2019).

**Figure 1:**
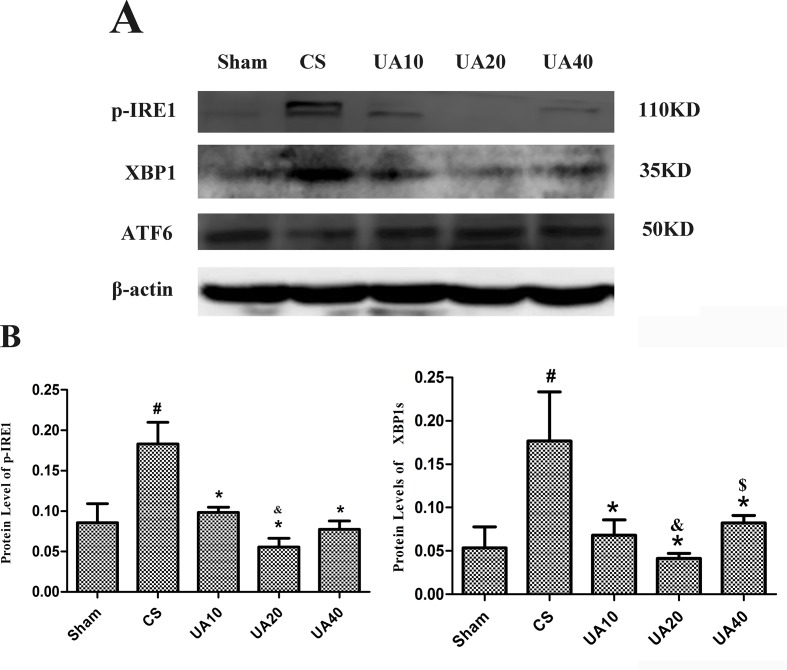
**(A)** Ursolic acid on CS-induced IRE1 and ATF6 activation. Expression of key molecules in the IRE1 and ATF6 pathways in CS-induced emphysema model with and without ursolic acid intervention was assessed by Western blot. **(B)**. Integrated optical density (IOD) of protein expression. ^#^Compared with Sham group, *p* < 0.05, *Compared with CS group *p* < 0.05, ^&^Compared with UA10 group *p* < 0.05. ^$^Compared with UA20 group *p* < 0.05.

Our previous work confirmed that PERK pathway mediating ERS associated cell apoptosis participated in CS induced emphysema in rats (Lin et al., 2017). IRE1 pathway was activated as well; nevertheless, ATF6 pathway seemed not involved (**Figure 1**). The active phases, durations, variation tendencies, and effects of the 3 UPR pathways still need to be clarified. It has been reported that intraperitoneal injection of CSE for 3 weeks could induce emphysema in SD rats (Hanaoka et al., 2011). We use this CSE induced emphysema rat model to carry out following researches.

In addition, CS-induced oxidant stress and cell apoptosis could promote TGF-β1 secretion, one of the main inducers of cell proliferation and airway-vessel remodeling in COPD (Ichimaru et al., 2012; Soltani et al., 2012; Soltani et al., 2016; Mahmood et al., 2017; Guan et al., 2017). Among its downstream pathways, TGF-β1/Smad2.3 signaling pathways facilitate epithelial mesenchymal transition (EMT) and endothelial mesenchymal transition (EndMT), key participants in airway-vessel remodeling in COPD (Soltani et al., 2012; Mahmood et al., 2017; Sohal, 2017).

Ursolic acid (UA) is a pentacyclic triterpenoid compound widely distributed in natural plants, such as apple peels, herbal medicines, and many other edible plants (Cui et al., 2006). UA has recently gained increasing attention due to its multiple beneficial effects including antioxidant (Tsai and Yin, 2008), anti-inflammatory (Li et al., 2015), antitumor (Prasad et al., 2016), and anti-apoptotic effects (Yang et al., 2014). UA has already been used in Phase I clinical trial for solid tumors (Zhu et al., 2013; Qian et al., 2015). Researchers reported previously that UA could downregulate PERK pathway and alleviate ERS associated apoptosis in heat stress induced mouse cardiac myocytes apoptosis (Yang et al., 2014). We found in our previous work that UA could alleviate CS induced emphysema partly through UPR-PERK and IRE1 pathways (Lin et al., 2017) (**Figure 1**). UA is also an antagonist of TGF-β1 (Murakami et al., 2004); however, whether and when UA could improve CSE-induced emphysema and airway remodeling, and the potential underlying mechanisms, deserve further exploration.

## Methods

### Compounds and Reagents

Antibodies against eIF-2a and CHOP were obtained from Santa Cruz Biotechnology (Santa Cruz, USA). Antibodies against p-eIF2α, Bcl-2, Bax, Caspase12, and p-IRE1 were purchased from Abcam Technology (Cambridge, UK). Antibodies against PERK, p-PERK, Cleaved-Caspase3, Caspase8, and Caspase9 were purchased from Cell Signaling Technology (Denver, USA). Antibodies against ATF6 were purchased from Wanlei Biology Technology (Shenyang, China). Cigarettes were acquired from Marlboro, USA. UA was bought from Wanxianghengyuan Biotechnology (Tianjin, China), and dissolved in phosphate-buffered saline (PBS) with 1% Tween80 before use. The Bicinchoninic acid (BCA) protein assay kit was acquired from Pierce (Thermo scientific, Rockford, USA), and the super enhanced chemiluminescence kit was purchased from Applygen Technologies Inc. (Beijing, China).

### Cigarette Smoke Induced Emphysema Model

Briefly, rats were randomized into one of five treatment groups (N = 10 each): Sham, CS, UA10, UA20, and UA40. UA rats were administrated 10, 20, or 40 mg/kg body weight UA via gavage 30 min before the first CS exposure every day. Sham and CS rats were given vehicle instead. CS and UA rats were exposed to smoke of 16 filters removed 1R3F cigarettes for 30 min a time, two times a day, 6 days a week, for 3 months. Rats in the Sham group were exposed to normal air.

### Cigarette Smoke Extract Preparation (Chen et al., 2009; Hanaoka et al., 2011)

CSE was prepared as previously reported (Chen et al., 2009; Hanaoka et al., 2011). Briefly, one unfiltered cigarette was burned and the smoke was delivered to the subsurface of PBS (1 ml per cigarette) using a vacuum pump. Particles and bacteria were removed using a 0.22-μM filter. The CSE-PBS solution (pH 5.2–5.3) was prepared fresh before each use.

### Cigarette Smoke Extract Induced Emphysema Model (Hanaoka et al., 2011)

One hundred eight 6-week-old male SD rats were maintained with a standard laboratory diet and free access to water. After a 2-week adaptation period, the rats were randomly assigned into three intervention groups (n = 36 each): control group (Sham), CSE group (CSE), and UA 20 mg/kg group (UA). Rats in each group were further divided into three subgroups: 2-week group (2W), 3-week group (3W), and 4-week group (4W) (n = 12 each). Rats in the CSE and UA groups were intraperitoneally injected with 1 ml CSE-PBS solution on days 1, 8, 15, and 21. Rats in the Sham group were intraperitoneally injected with 1 ml PBS. Rats in the UA group were administered 20 mg/kg UA intragastrically daily in a volume of 3 ml. Rats in the Sham and CSE groups were administrated an equal volume of vehicle. The body weights of rats were measured.

### Collection of Blood and Lung Specimens

The rats’ chests were opened and blood was drawn from the inferior vena cava. The left lung was inflated with 0.5% low-melting agarose at a constant pressure of 25 cm H_2_O, and fixed in 10% formalin for 48 h. The paraffin-embedded sections of the left lung were used for histopathological examination. The right lung was frozen in liquid nitrogen for 5 min before storing in −80°C freezer for further Western blot analysis.

### Histopathological Assessment of the Degree of Emphysema and Airway Remodeling

To assess the morphological changes that characterize emphysema and airway remodeling in the experimental rat lungs, the mean linear intercept (MLI), mean alveolar number (MAN), and bronchial wall thickness were measured in lung tissue sections by hematoxylin and eosin (HE) staining. Lung tissue sections were examined by light microscopy at 50× and 100× magnification. Inter-alveolar wall distance was measured by MLI, defined as the total length of the test line divided by the number of intersected alveolar walls (Xu et al., 2004). Alveolar density was measured by MAN, which was defined as the number of alveoli in each field (Xu et al., 2004). The bronchial wall thickness was evaluated under light microscope at 100× magnification. Four slices, without cartilage ring intact bronchial tracheal transection, were randomly selected from each rat; the minimum/maximum diameter of airways was 0.5, in order to access the same level of airway. Image Pro Plus5.0 analysis software was used to measure the tracheal basement membrane perimeter (Basement membrane perimeter, Pbm) and tracheal wall area (total wall area, Wat). The Wat/Pbm value of each trachea was calculated, and the average value was used to compare the airway wall thickness of rats in different groups. For each sample, MLI and MAN were calculated in four random fields without any airway structure.

### Immunohistochemical Analysis

Selected samples were embedded in paraffin, sectioned (4-μm thickness), paraffinized, and rehydrated. Then antigen retrieval was performed using a microwave in 10 mM citrate buffer (pH 6). Endogenous peroxidase was blocked with 3% peroxide for 5 min and then incubated with 10% unimmunized serum. Sections were incubated with primary 8-OHdG (1:500), Cleaved-Caspase3 (1:500), p-Smad2 (1:500), TGF-β1 (1:500), and α-SMA (1:1,000) antibodies overnight at 4°C, then washed and incubated with the secondary antibody (1:1,000) for 1 h at room temperature. Staining was developed by incubating with Diaminobenzidine (DAB) and DAB enhancer. Protein expression levels were measured using Image Pro Plus software. One field in a different quadrant of each section was assessed.

### Western Blot Analysis

Whole cell lysates were prepared from the rat lung tissues. Briefly, lung tissues were resuspended in an ice-cold lysis buffer containing 50 mM Tris–HCl (pH 7.4), 150 mM NaCl, 1% NP-40, 0.1% SDS, 1 mM protease inhibitor cocktail and protein phosphatase inhibitor (Roche Applied Science, USA) and then homogenized for 15 s at 4°C four to five times. Cell lysates were centrifuged at 12,000 × *g* for 30 min at 4°C to remove cellular debris. The protein concentration was determined using BCA protein assay kit. Equal amounts of proteins (20–60 μg) were separated on 8–10% SDS-PAGE gels and transferred to PVDF membranes (Merck Millipore, USA). The membranes were blocked in 5% non-fat milk at room temperature for 2 h, and then incubated with diluted primary antibodies overnight at 4°C. Blots were stripped and re-probed with anti-β-actin antibody to demonstrate equal loading. After incubation with horseradish peroxidase (HRP)-conjugated secondary antibody, the chemiluminescent signal was detected using the Super Enhanced Chemiluminescence Kit. Band density was quantified using Quantity One software (Bio-Rad Laboratories, USA).

### Statistical Analysis

Data were represented as the Mean ± SD. SPSS13.0 software and Graph Pad Prism 5.0 were used for statistical analysis. One-way ANOVA was used to compare differences among groups. P-value of <0.05 was considered to indicate statistical significance.

## Results

### Ursolic Acid Alleviated the Activation of IRE1 Pathway in Cigarette Smoke Induced Emphysema Rat Lung

We used Western blot method to assess the activation of IRE1 and ATF6 pathways in CS induced emphysema rat lung. Our results demonstrated that CS exposure for 3 months up-regulated protein expression of phosphorylated IRE1(p-IRE1), as well as XBP1 molecule that functions downstream. UA administration alleviated the activation of IRE1 pathway. However, the ATF6 pathway seem to not be activated. (**Figure 1**).

### Ursolic Acid Prevented Body Weight Loss in Cigarette Smoke Extract Treated Rats

**Figure 2:**
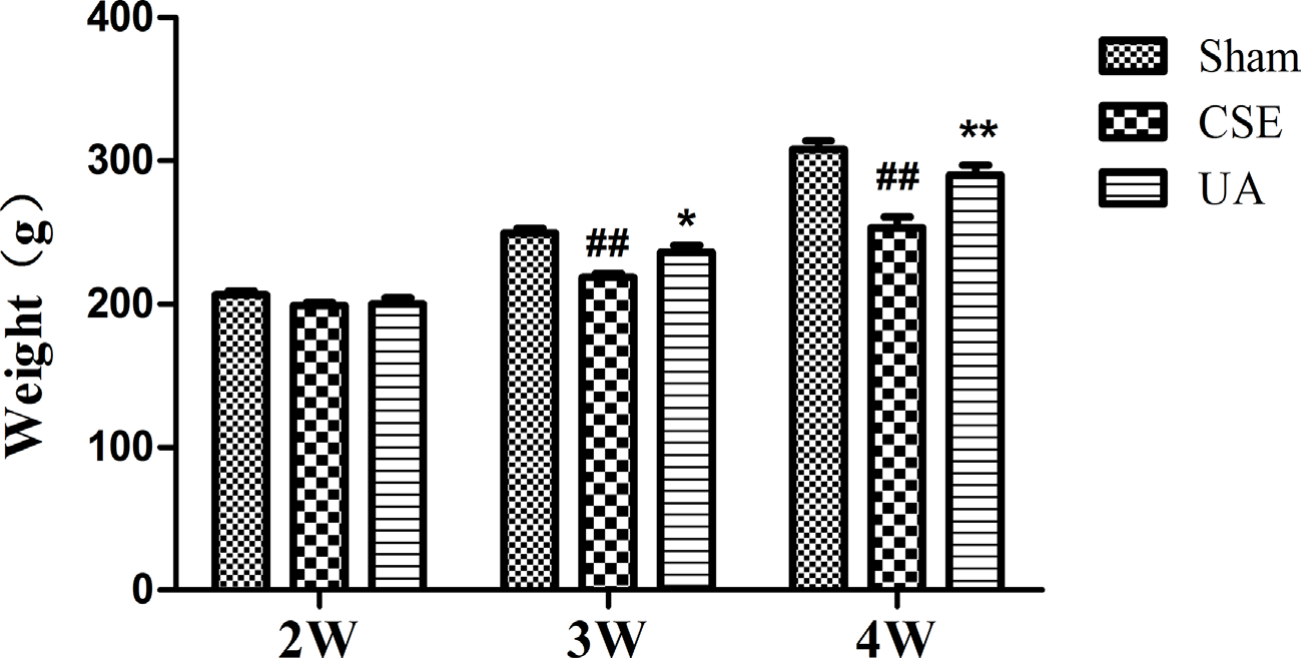
Body weight of rats. ^##^Compared with the corresponding Sham group, p < 0.01; *compared with corresponding CSE group, p < 0.05; **compared with corresponding CSE group, p < 0.01.

Emaciation is an important complication and an indicator for severity and poor prognosis of COPD. Rats that received peritoneal injection of CSE for 3 or 4 weeks weighed less than those in the corresponding Sham groups. UA alleviated the degree of weight loss-induced in rats exposed to CSE for 3 or 4 weeks (**Figure 2**).

### UA Improved Cigarette Smoke Extract-Induced Emphysema in Rats

**Figure 3:**
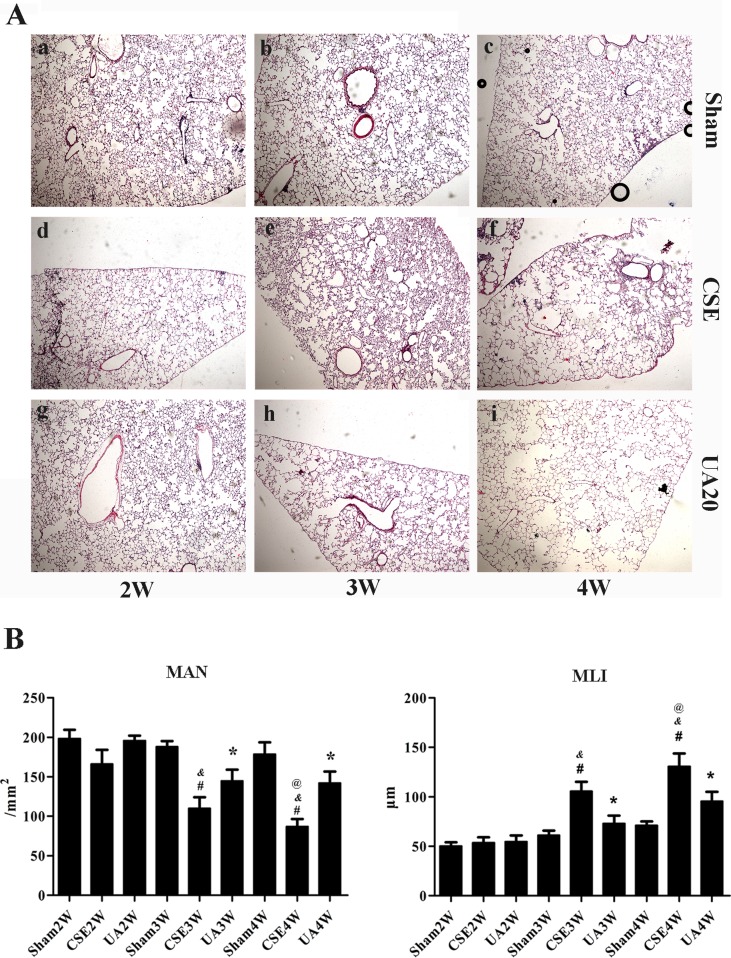
Ursolic acid alleviates CSE-induced emphysema in rats. **(A)** Histopathological analyses of rat lung sections with H&E staining (×50). a, Sham2W; b, Sham3W; c, Sham4W; d, CSE2W; e, CSE3W; f, CSE4W; g, UA2W; h, UA3W; I, UA4W. **(B)** Morphometric measurements of MLI and MAN in lung tissue sections with HE staining. MLI level was significantly increased, while MAN level was significantly reduced in CSE3W and CSE4W compared to Sham groups at the same time point. Ursolic acid improved CSE induced changes above. ^#^ *p* < 0.05 compared with corresponding Sham group, *p < 0.05 compared with corresponding CSE groups, ^&^p < 0.05 compared with CSE2W group, ^@^p < 0.05 compared with CSE3W group.

Tissue morphological analyses were performed to determine the protective effect of UA on CSE-induced emphysema in rats. We found that peritoneal injection of CSE for 3 weeks induced emphysema. Alveolar structure appeared disorganized, alveolar walls were thinned or ruptured, fused into bullae, and pulmonary fibrosis was observed. More severe emphysema was observed in rats administered CSE for 4 weeks. UA improved CSE-induced emphysema of rats (**Figure 3A**). MAN and MLI were further applied to evaluate the severity of rat emphysema in different groups. MLI was significantly increased and MAN was significantly decreased after injection of CSE for 3 weeks, as evidenced by HE staining. These changes were more severe in animals administered CSE for 4 weeks. UA treatment for 3 or 4 weeks improved CSE-induced changes in MAN and MLI (**Figure 3B**).

### Ursolic Acid Inhibited Cigarette Smoke Extract Activated Unfolded Protein Response Pathway

**Figure 4:**
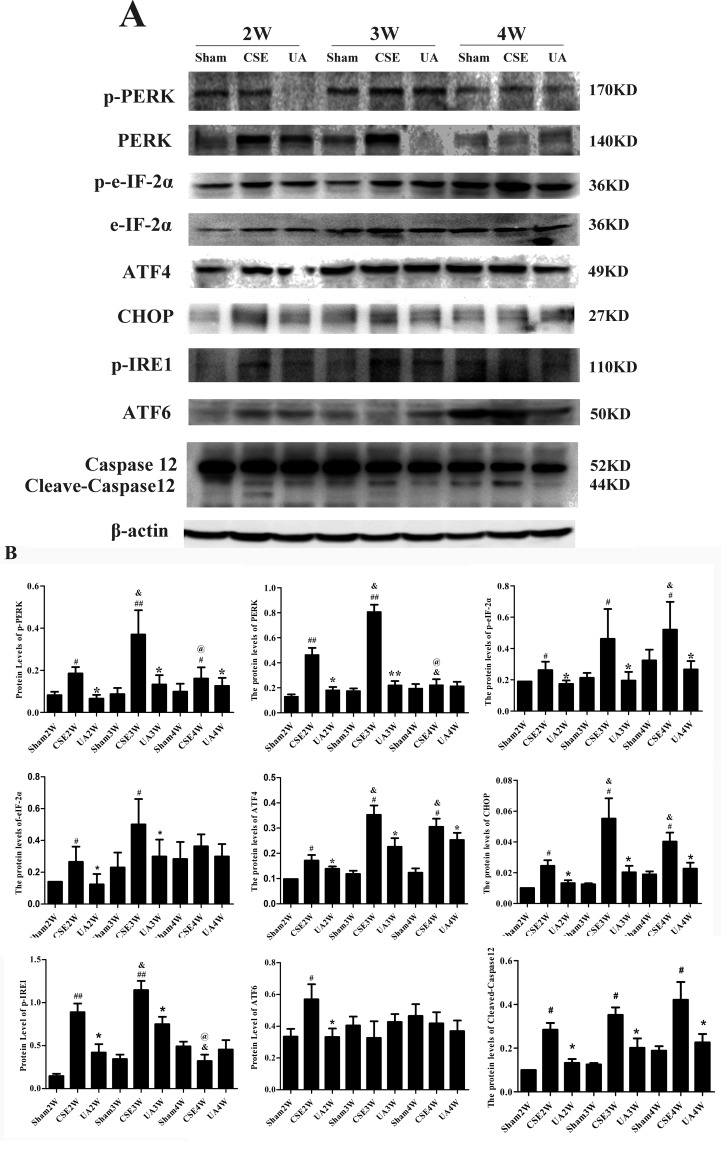
Expression of the key factors in the unfolded protein response pathways and Cleaved-Caspase12. **(A)** Protein expression in lungs of rats by Western bolt analyses. **(B)** IOD indicating protein expression level. ^#^ *p* < 0.05 compared with Sham groups at the same time points, ^##^ *p* < 0.01 compared with Sham groups at the same time points, *p < 0.05 compared with CSE groups at the same time points. **p < 0.01 compared with CSE groups at the same time points, ^&^ *p* < 0.05 compared with CSE2W group. ^@^ *p* < 0.05 compared with CSE3W group.

Expression and activation of initiators and key factors in the UPR pathways, and an ERS-specific apoptosis molecule, Cleaved-Caspase12, were measured in rat lungs by Western blot (**Figure 4**).

PERK and eIF2α expressions were upregulated in CSE2W and CSE3W groups. P-PERK and p-eIF2α, ATF4 and CHOP expressions were increased in all CSE groups. P-PERK and PERK expressions were highest in the CSE3W group, while p-eIF2α expression was highest in CSE4W group. The ATF4 and CHOP expressions were higher in CSE3W and CSE4W groups than in CSE2W group. CSE-induced PERK-CHOP pathway activation was inhibited by UA treatment.

Injection of CSE for 2 weeks increased expression of p-IRE1, and injection for 3 weeks further enhanced p-IRE1 expression. UA treatment alleviated CSE-induced phosphorylation of IRE1.

Injection of CSE for 2 weeks increased expression of activated ATF6 (50KD), UA reduced expression of activated ATF6 in rat lungs in the CSE2W group. Injection of CSE for 2, 3, and 4 weeks all could increase expression of Cleaved-Caspase12. UA alleviated the activation of Caspase12 in rat lungs.

### Ursolic Acid Alleviated Cigarette Smoke Extract-Induced Activation of Intrinsic and Extrinsic Apoptotic Pathways

**Figure 5:**
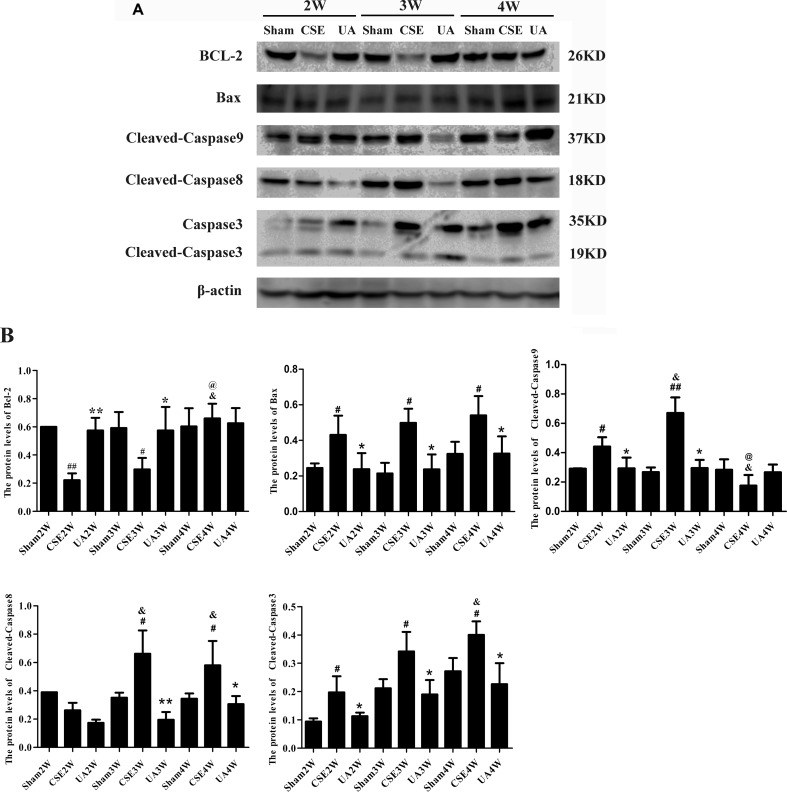
Expression of intrinsic and extrinsic apoptosis-related molecules in the lungs of rats exposed to CSE. **(A)** Protein expression in rat lungs was assessed by Western bolt. **(B)** The IOD value of proteins expression. ^#^ *p* < 0.05 compared with Sham groups at the same time points, ^##^ *p* < 0.01 compared with Sham groups at the same time points, *p < 0.05 compared with CSE groups at the same time points, **p < 0.01 compared with CSE groups at the same time points, ^&^ *p* < 0.05 compared with CSE2W group, ^@^ *p* < 0.05 compared with CSE3W group.

The intrinsic and extrinsic apoptotic pathways indicators were activated by CSE injection, but timing and duration differed between groups, while UA administration alleviated the upregulation (**Figure 5**).

Expression of Bcl-2 was down-regulated in CSE2W and CSE3W groups, and UA treatment ameliorated CSE-induced Bcl-2 down-regulation.

Bax expression was up-regulated in all CSE groups, and UA treatment down-regulated CSE-induced Bax expression.

Caspase9 was activated by CSE in CSE2W and CSE3W groups, Cleaved-Caspase9 was more strongly induced in CSE3W than CSE2W animals. UA could attenuate the up-regulation of Cleaved-Caspase9.

Caspase8 was activated by CSE in CSE3W and CSE4W groups, UA attenuate the up-regulation of Cleaved-Caspase8.

Caspase3 was activated by CSE in CSE2W, CSE3W and CSE4W groups, Cleaved-Caspase3 was more strongly induced in CSE4W than CSE2W animals. UA attenuate the up-regulation of Cleaved-Caspase 3 expression.

### UA Alleviated Cigarette Smoke Extract-Induced Airway Remodeling through Downregulation of p-Smad2 and p-Smad3 Molecules

**Figure 6:**
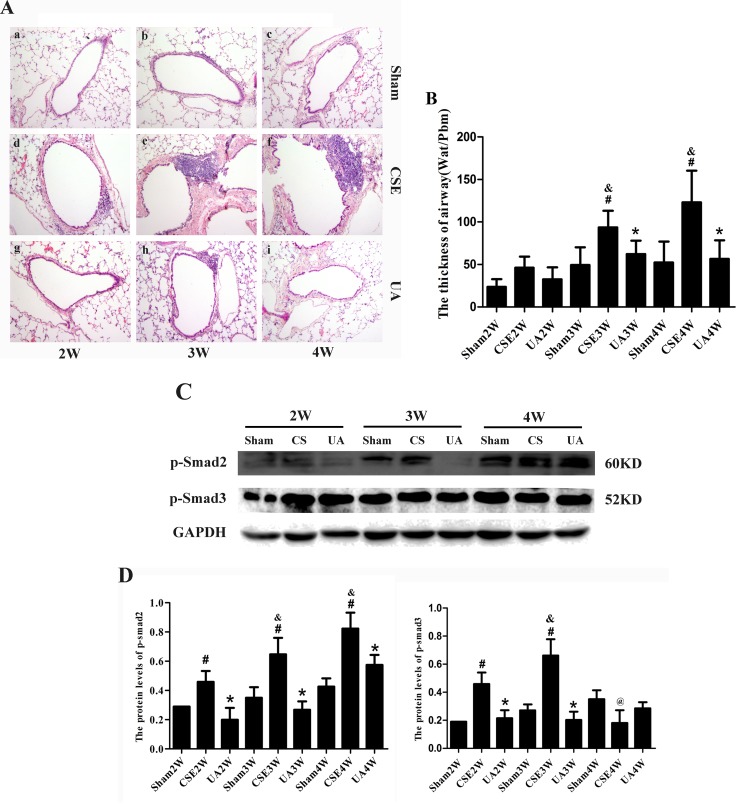
Airway remodeling-associated changes, p-Smad2 and p-Smad3 expression in the lungs of rats exposed to CSE. **(A)** Airway and the tissue surrounding tissues stained with HE (×100). **(B)** The Thick of airway (Wat/Pbm). ^#^ *p* < 0.05 compared with corresponding Sham groups at the same time points, *p < 0.05 compared with CSE groups at the same time points. **(C)** Protein expression of p-Smad2 and p-Smad3 in rat lung. **(D)** The IOD value of proteins expression. ^#^ *p* < 0.05 compared with corresponding Sham group, *p < 0.05 compared with CSE groups at the same time points, ^&^ *p* < 0.05 compared with CSE2W group, ^@^ *p* < 0.05 compared with CSE3W group.

Peritoneal injections of CSE for 3 or 4 weeks induced apparent airway remodeling-associated changes. Airway walls were thickened, inflammatory cells infiltrated into the airway wall, and collagen deposition was increased around the airway. UA administration alleviated CSE induced changes above (**Figure 6A and B**).

In the CSE2W, CSE3W, and CSE4W groups, expression of p-Smad2 was upregulated in the lungs, an affect ameliorated by UA. P-Smad3 expression was upregulated in CSE2W and CSE3W groups, and UA administration alleviated CSE induced changes above (**Figure 6C and D**). Thus, UA attenuated CSE-induced airway-remodeling partly by reducing activation of Smad2 and Smad3 molecules.

## Discussion

It is estimated that COPD will be the third leading cause of death worldwide by 2020 (Angelis et al., 2014), but, due to its high prevalence and chronicity, management of COPD is costly. However, current therapeutic interventions may not successfully reverse airway remodeling and emphysema in COPD. Thus, effective interventions to prevent COPD progression are urgently sought.

Recently, increasingly data suggests that imbalanced proliferation and apoptosis of different lung cells might contribute to the pathogenesis of COPD (Calabrese et al., 2005; Demedts et al., 2006; Yu and Guo, 2007; Sohal, 2017; Guan et al., 2017), causing damage in airway-vessel and lung tissue structure, and subsequent development of airway-vessel remodeling and emphysema (Demedts et al., 2006; Henson et al., 2006; Yamada et al., 2016). The intrinsic, extrinsic and ERS-associated apoptotic pathway were implicated in apoptosis in COPD (Lin et al., 2015; Lin et al., 2017). However, the precise mechanisms underpinning this process are not yet clear.

The ER (endoplasmic reticulum) is an organ responsible for protein synthesis and maturation. The accumulation of unfolded proteins in the ER lumen activate a series of UPR signaling cascades in response to cellular stress such as oxidant stress, hypoxia, and high-temperature exposure (Banerjee et al., 2017). These responses protect cells from ERS by promoting protein folding, degradation of misfolded protein and inhibition of global protein synthesis (Xu et al., 2005; Ron and Walter, 2007). However, excessive ERS can lead to programmed cell death through an ERS-associated apoptosis pathway and activation of Caspase 12 in rodents (Caspase 4 in human) (Kaufman, 2002; Ron, 2002; Ron and Walter, 2007). Several studies have reported CSE-induced apoptosis *in vitro*, especially ERS associated cell apoptosis (Lee et al., 2017a; Lee et al., 2017b; Jiao et al., 2017; Song et al., 2017; Iu et al., 2017). This process involves UPR signaling pathways activation, induced *via* ER transmembrane molecules, including PERK, IRE1 and ATF6, and sequence different signaling pathways, final activating ERS-associated apoptosis (Tokunaga et al., 2010; Gan et al., 2011; Tagawa et al., 2011; Yuan et al., 2012; Kitaguchi et al., 2012; Kelsen, 2016). Of the three UPR signaling cascades involved in emphysema, the mechanism of PERK in emphysema is most clear (Gan et al., 2011). However, the expression and activation of IRE1 and ATF6 pathways in CS-induced emphysema has not been reported previously. Our work showed that in rats the IRE1 pathway of UPR is up-regulated in CS induced-emphysema, however, ATF6 expression is not. UPR also involves in mediating the activation of intrinsic and extrinsic apoptotic pathways. However, the exact mechanism in CSE induced emphysema has not been illuminated.

TGF-β1 is a pro-fibrotic factor that regulates expression of extracellular matrix proteins such as procollagen I and III, fibronectin, versican, and tenascin (Aschner and Downey, 2016). Variation in the TGF-β1 encoding gene has been suggested to be one of the genetic determinants of COPD (Hogg et al., 2004; Zhang et al., 2011; Tang et al., 2016; Xu et al., 2017; Godinas et al., 2017). Previous reports have also shown TGF-β1 expression to be increased in airway epithelium, airway smooth muscle, and macrophages in the lungs of COPD patients (de Boer et al., 1998; Takizawa et al., 2001; Gohy et al., 2014). TGF-β1 exerts its effects by regulating its downstream transcription factor Smads. TGF-β1/Smad2.3 signaling was reported to play important roles in CS-induced airway remodeling of COPD (Soltani et al., 2012; Xu et al., 2012).

In the current study, we found that injections of CSE for 3 or 4 weeks could induce significant emphysema, and pathologic changes were more severe in CSE4W group. In that case, we call pathological changes in CSE2W, CSE3W, CSE4W rat pre-emphysema, middle-emphysema, severe-emphysema, respectively. ERS specific apoptotic indicator Cleaved-Capase12 was activated in all CSE groups, and thus ERS-associated apoptosis may exert its action in all stages of CSE-induced emphysema. Intrinsic apoptotic index Cleaved-Caspase9 was upregulated in CSE2W and CSE3W groups, while, extrinsic apoptotic index Cleaved-Caspase8 was upregulated in CSE3W and CSE4W groups. Communal downstream indicators of apoptosis Cleaved-Caspase3 expression was upregulated in CSE2W, CSE3W, and CSE4W group. CSE-induced expression of three transmembrane molecules (PERK, IRE1, and ATF6) associated with ERS. However, the phase and duration of these actions differed. PERK-CHOP pathway molecules were up-regulated in CSE2W, CSE3W, and CSE4W groups, which may indicate that the PERK pathway exerts its effect in all stages of CSE-induced emphysema through ERS-associated apoptosis, and the out-of-sync of the peak may be related to the activation order of the pathway and the presence of feedback effect (Liao et al., 2016). IRE1 was activated in CSE2W, and CSE3W groups, which may indicate that the IRE pathway exerts its effect in pre-emphysema and mild-emphysema through an ERS-associated program. ATF6 was activated in the CSE2W group, which may indicate that ATF6 pathway exerts its effect in pre-emphysema group. In short, CSE could activate all three apoptotic pathways, leading to apoptosis of pulmonary cells, and ultimately induction of emphysema and airway remodeling. However, the time of pathway activation varied between pathways. The activation duration of 3 pathways of UPR is also different. It seems that the IRE1 and ATF6 pathways of UPR were activated but then attenuated despite unresolved ERS, why?

Recently, researchers reported PERK shuts IRE1 down in the terminal stage of UPR, and that it exerts this effect through a phosphatase known as RPAP2 (RNA polymerase II-associated protein 2). RPAP2 reverses IRE1 phosphorylation, oligomerization, and RNase activation. This inhibits IRE1-mediated adaptive events, including activation of the cytoprotective transcription factor XBP1s, and ER-associated degradation of unfolded proteins. Furthermore, RIDD termination by RPAP2 unleashes DR5-mediated caspase activation and drives extrinsic cell death (Chang et al., 2018). In short, PERK attenuates IRE1 *via* RPAP2 to abort failed ER-stress adaptation and trigger apoptosis, which may explain our result that IRE1 pathway was only activated in early and middle stage of emphysema, and may participant in intrinsic apoptotic pathways, and at the severe or called end stage of emphysema, receptor mediate extrinsic apoptosis pathway activation may be due to the PERK and RPAP2 pathways.

UA intervention could alleviate the upregution of these three ERS transmembrane molecules and inhibit ERS-associated apoptosis in rat lungs. UA intervention alleviated CSE-induced activation of intrinsic and extrinsic apoptosis pathways also, through all these apoptotic pathways. UA alleviated CSE-induced lung cell apoptosis, and subsequently alleviated CSE-induced emphysema of rats.

CSE intervention also could upregulate p-Smad2 and p-Smad3 expression in the lungs of rats, implicating these proteins participated in CSE induced airway remodeling in the rat lungs. UA alleviated airway-vessel remodeling, partly through downregulating p-Smad2 and p-Smad3 expressions.

However, the failure to upregulation of ATF6 pathway remain to be investigated. Soon afterwards, we will discuss the interaction and the regulatory mechanisms of the three UPR pathways *in vitro* after CSE exposure.

## Conclusion

UA could alleviate CSE-induced emphysema and airway remodeling, a process involving all three UPR pathways. The process also involved suppression of ERS-associated apoptosis, attenuation of intrinsic and extrinsic apoptosis pathways, and down-regulation of p-Smad2/3.

## Ethics Approval

This study was approved by the ethics committee of the first affiliated hospital of Chinese Medical University.

## Author Contributions

QW participated in the design of the study and modification of the manuscript. JK conceived of the study and modified the manuscript. LL carried out the studies, performed the statistical analysis, and drafted the manuscript. GH and DH participated in its design and coordination, and performed the statistical analysis. All authors read and approved the final manuscript.

## Funding

This work is supported by grant from the National Key Technology Research and Development Program of the 13th National 5-year Development Plan (2016YFC1304103) and Liaoning Provincial Innovation Team Project (LT2013015).

## Conflict of Interest Statement

The authors declare that the research was conducted in the absence of any commercial or financial relationships that could be construed as a potential conflict of interest.
