# Supplementary figures and images for: Ursolic Acid Protected Lung of Rats From Damage Induced by Cigarette Smoke Extract
Source: Front Pharmacol. 2019 Jun 20;10:700. doi: 10.3389/fphar.2019.00700 (PMC6595172; doi:10.3389/fphar.2019.00700)

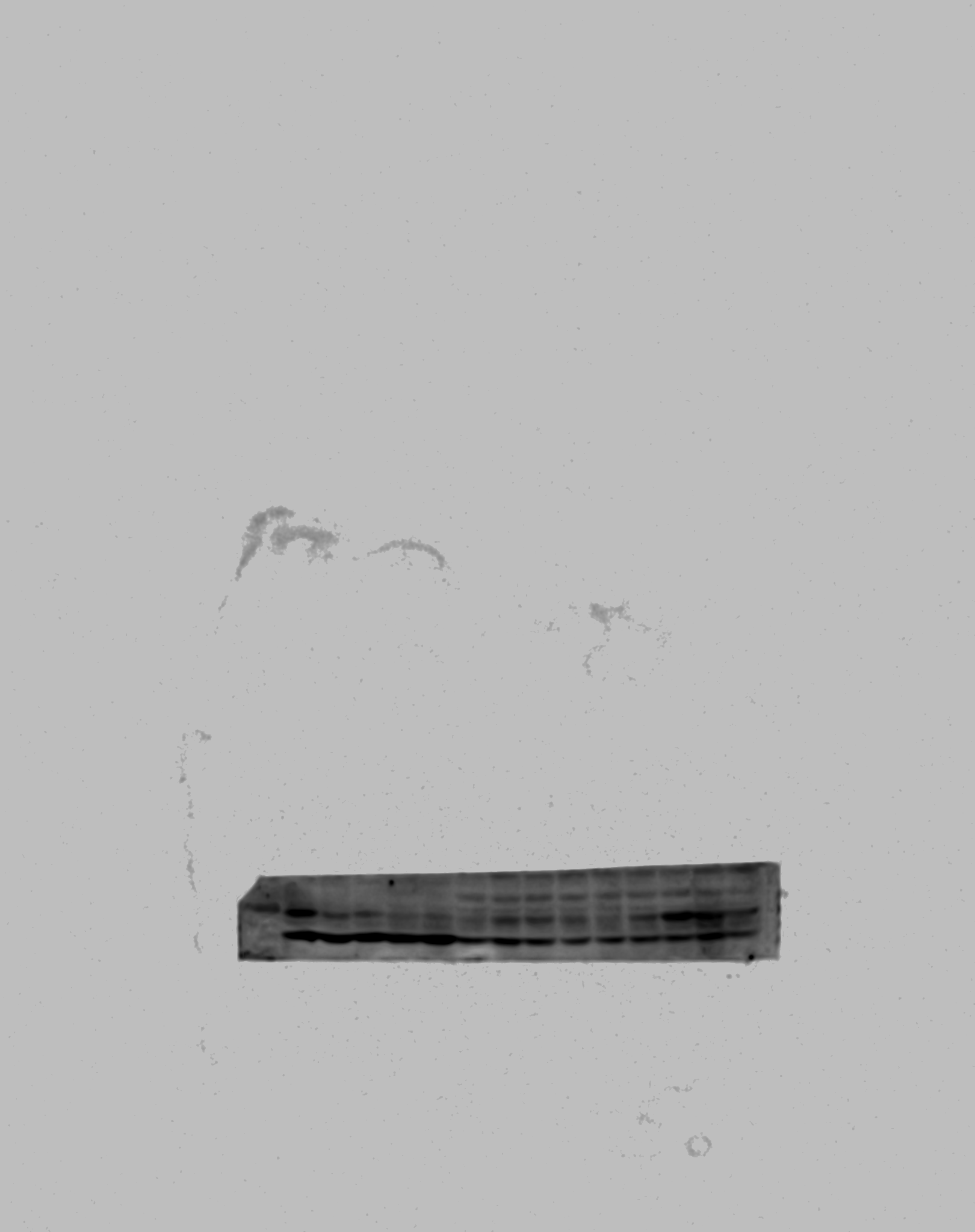

Supplement: Supplementary file 1 [file DataSheet_1.zip › supplementary materials-20190610/Figure4A-ATF6(50kd).bmp]

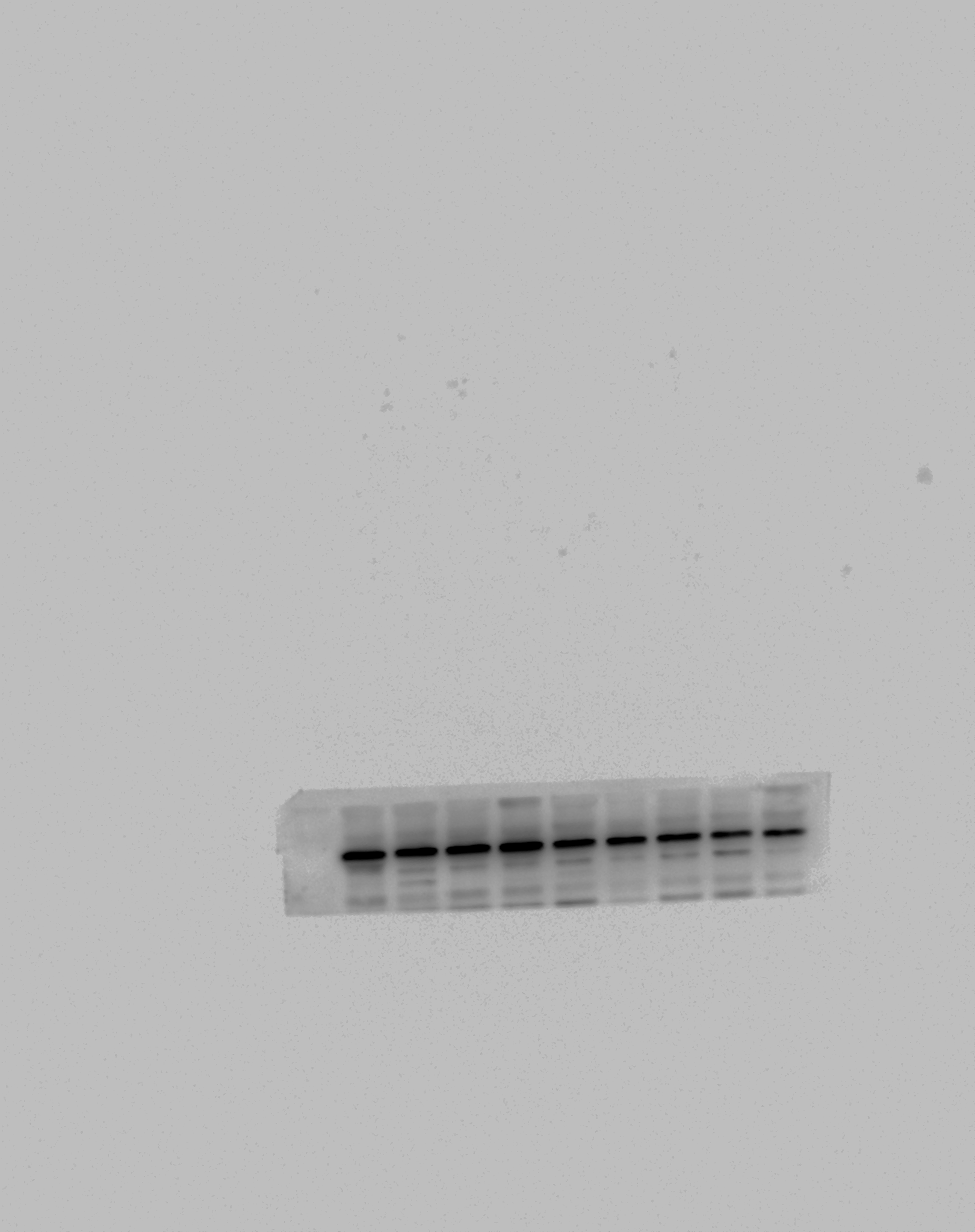

Supplement: Supplementary file 1 [file DataSheet_1.zip › supplementary materials-20190610/Figure4A-Casepase12.bmp]

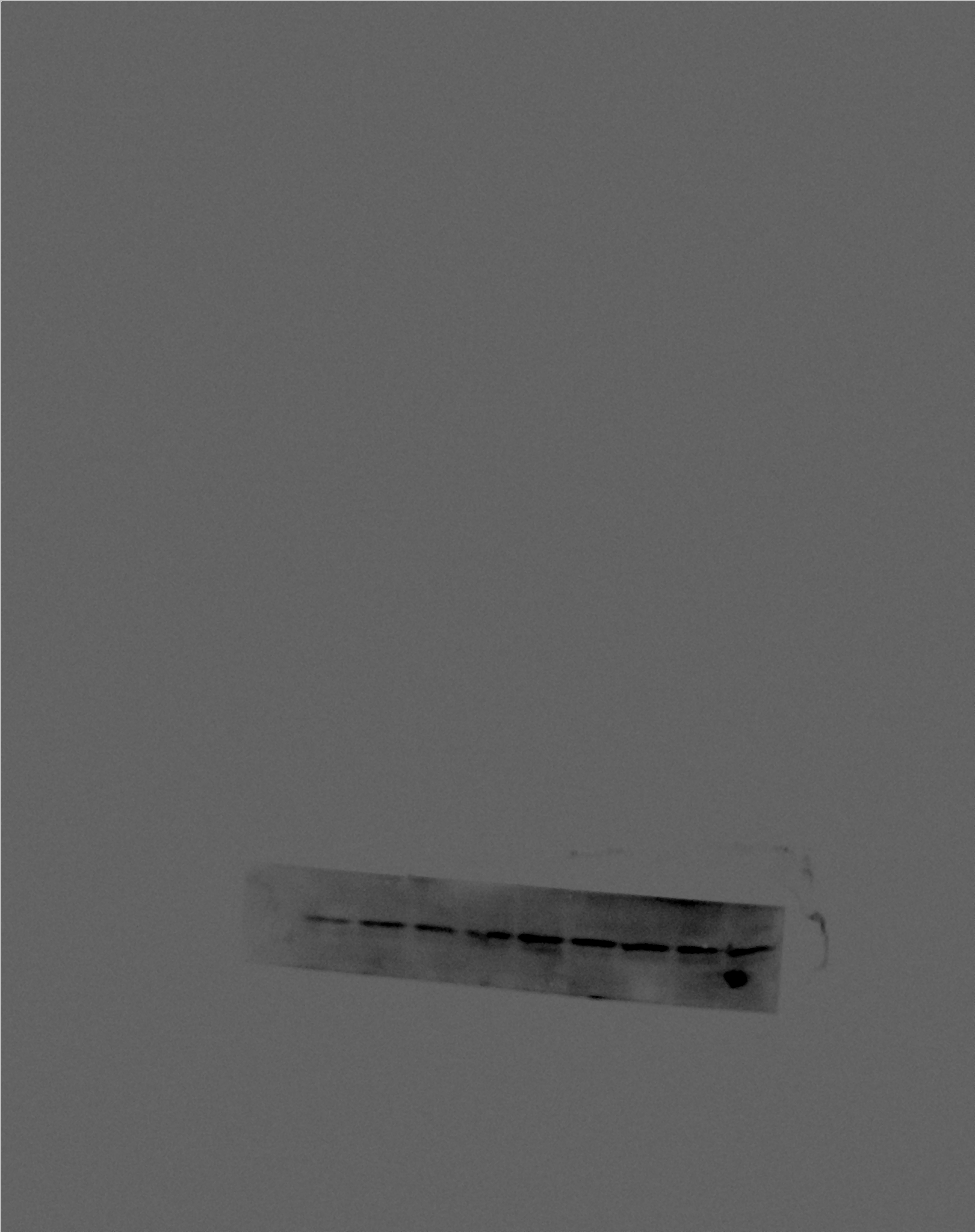

Supplement: Supplementary file 1 [file DataSheet_1.zip › supplementary materials-20190610/Figure4A-eIF-2a.bmp]

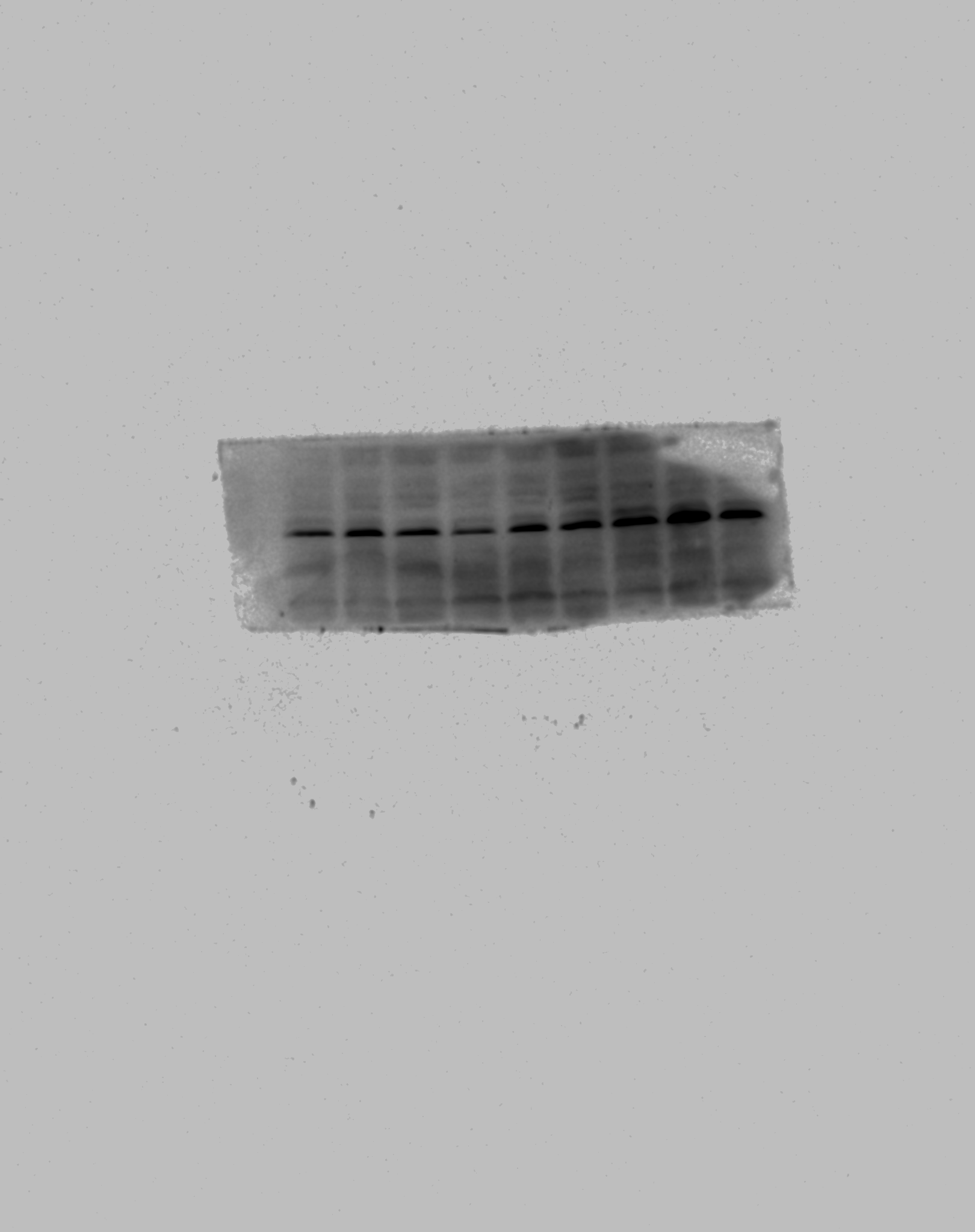

Supplement: Supplementary file 1 [file DataSheet_1.zip › supplementary materials-20190610/Figure4A-p-eIF-2a.bmp]

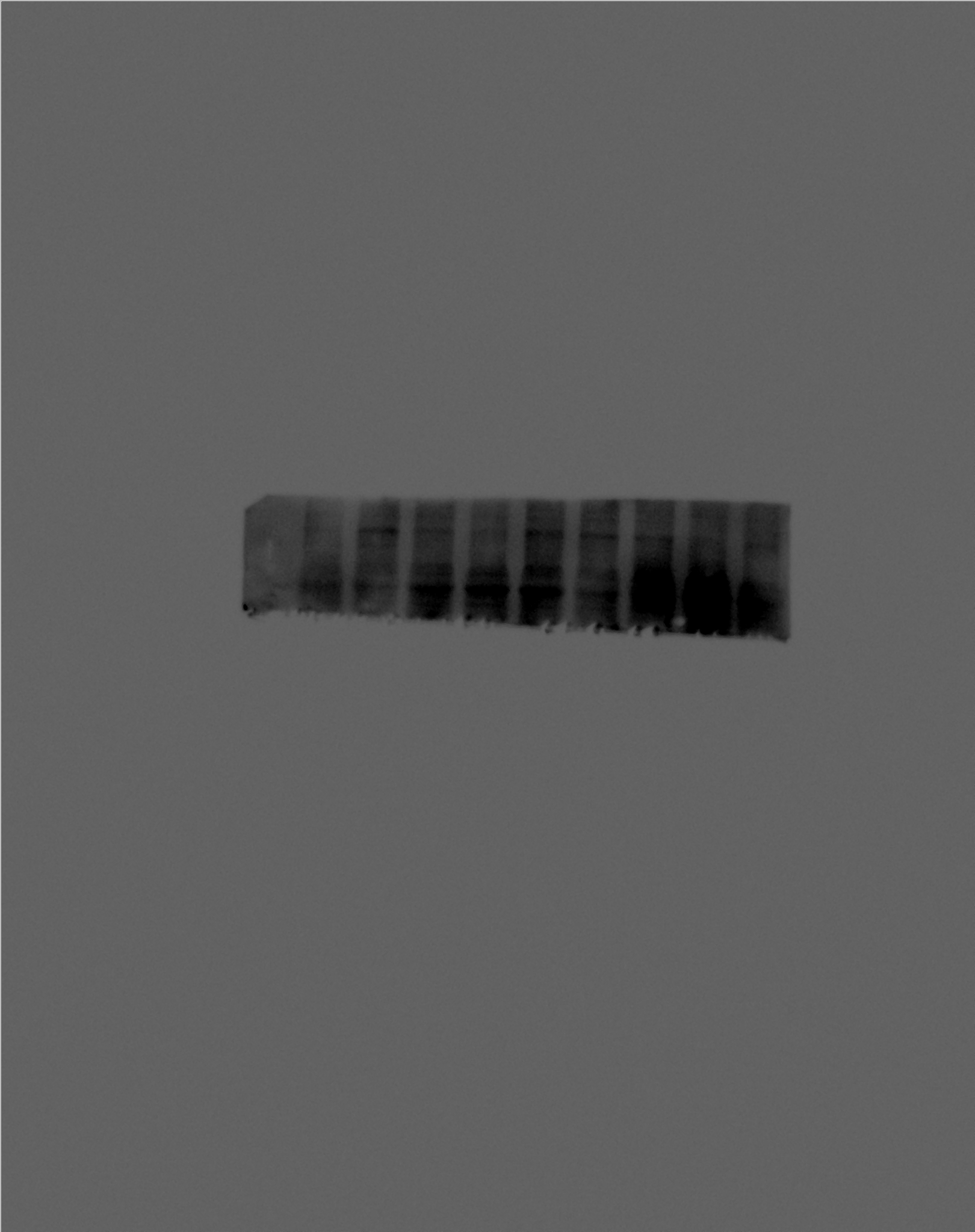

Supplement: Supplementary file 1 [file DataSheet_1.zip › supplementary materials-20190610/Figure4A-p-IRE1-2.bmp]

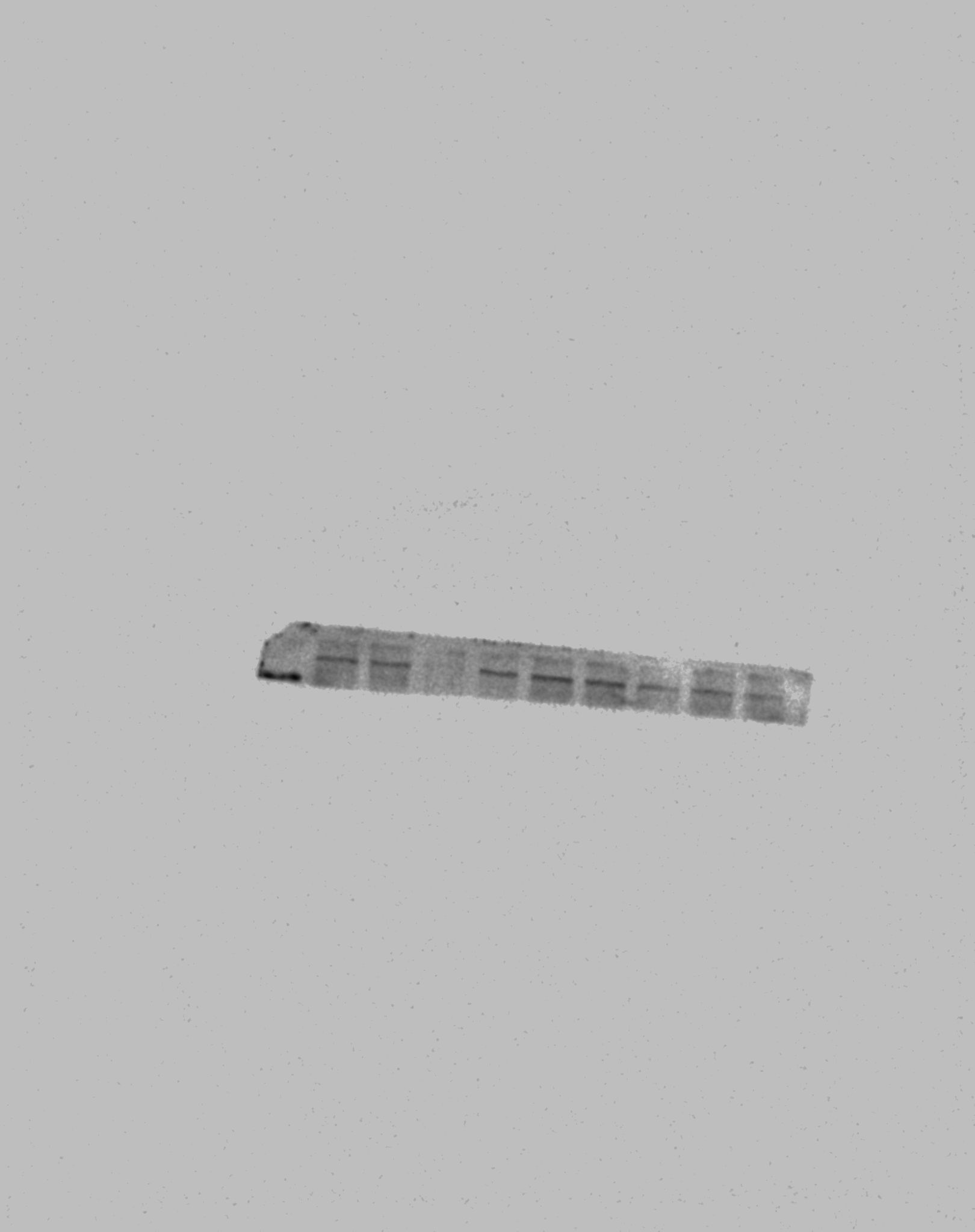

Supplement: Supplementary file 1 [file DataSheet_1.zip › supplementary materials-20190610/Figure4A-p-PERK.bmp]

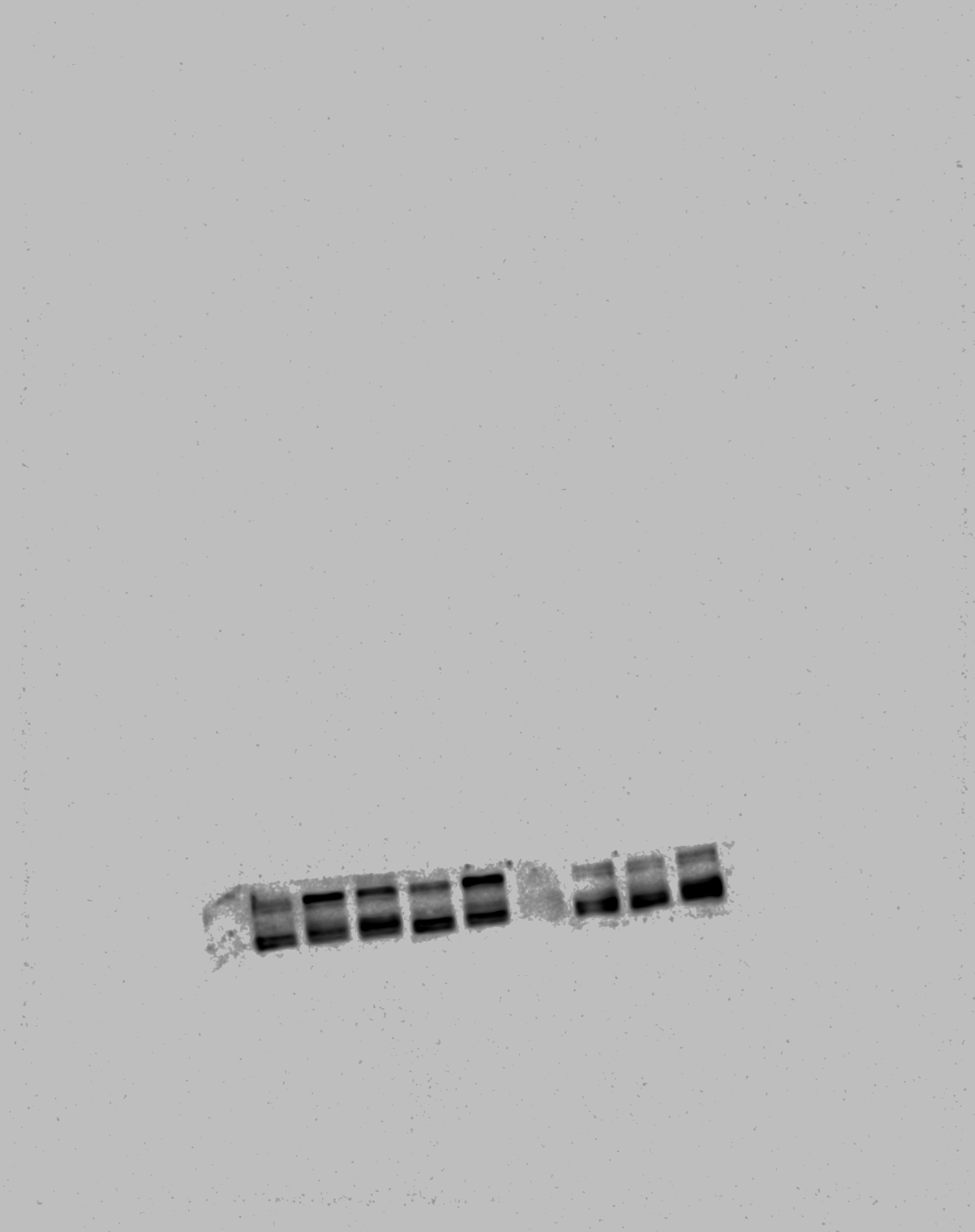

Supplement: Supplementary file 1 [file DataSheet_1.zip › supplementary materials-20190610/Figure4A-PERK.bmp]

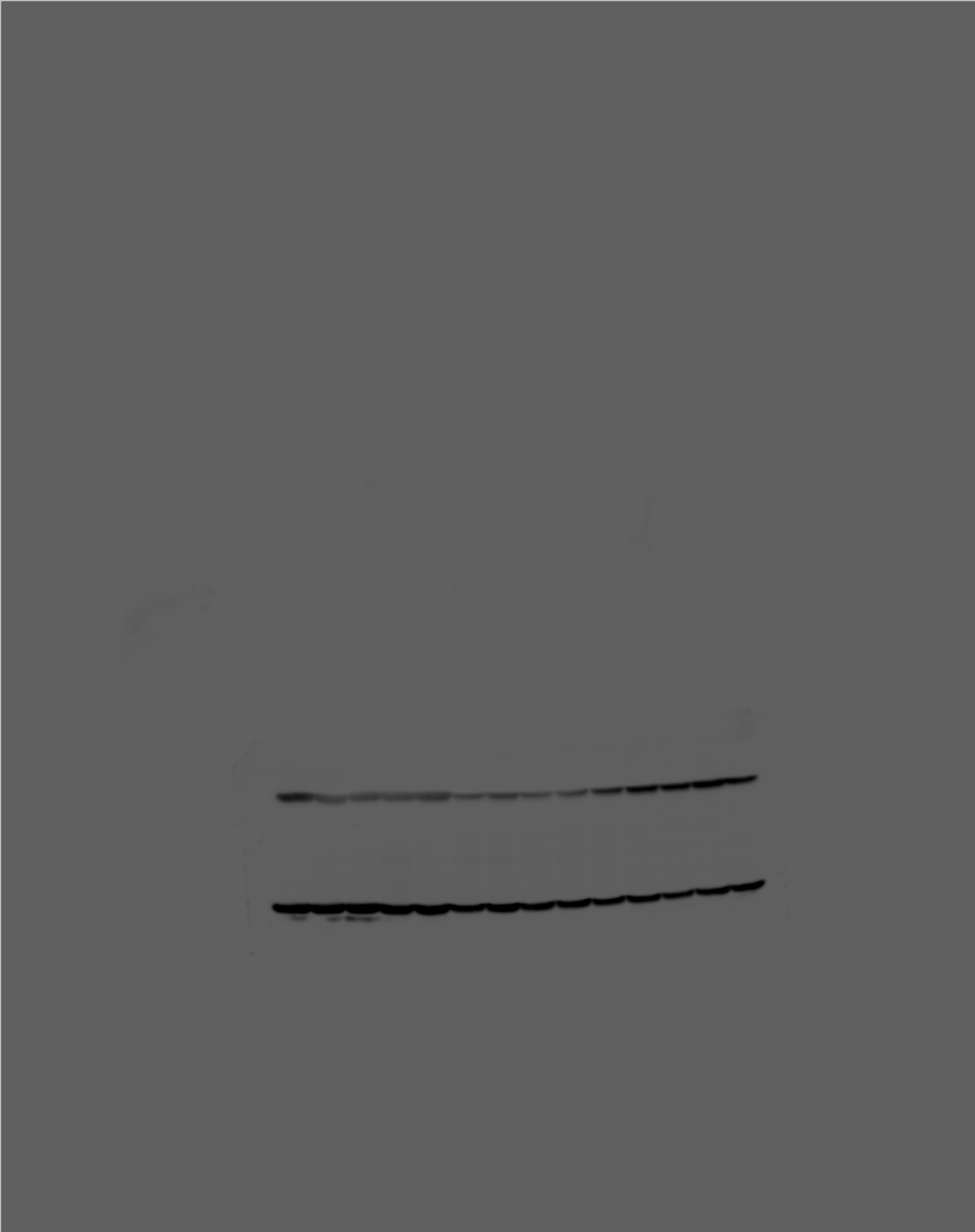

Supplement: Supplementary file 1 [file DataSheet_1.zip › supplementary materials-20190610/Figure4A-β-actin -Last 9 lanes(bottom).bmp]

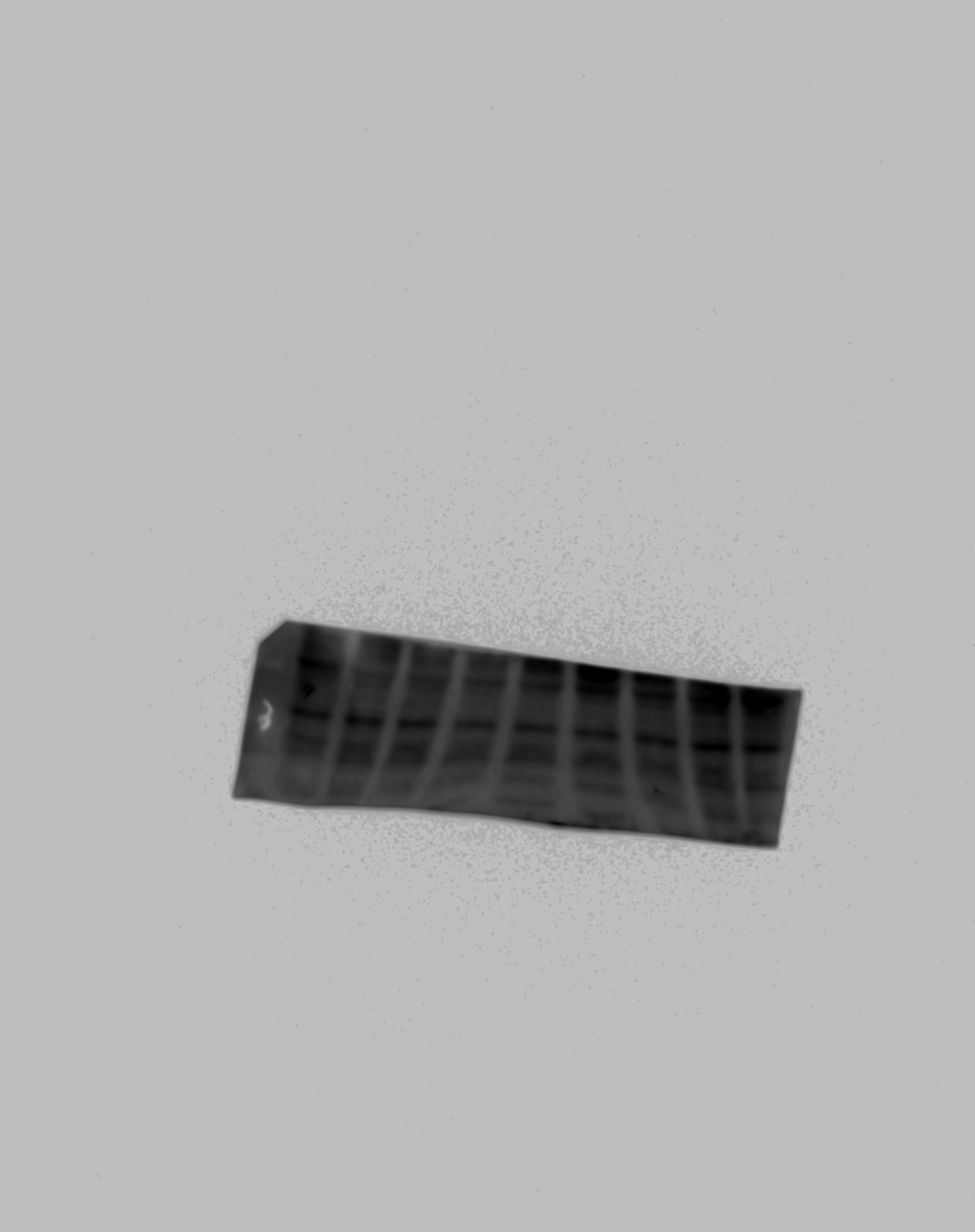

Supplement: Supplementary file 1 [file DataSheet_1.zip › supplementary materials-20190610/Figure5A-bax.bmp]

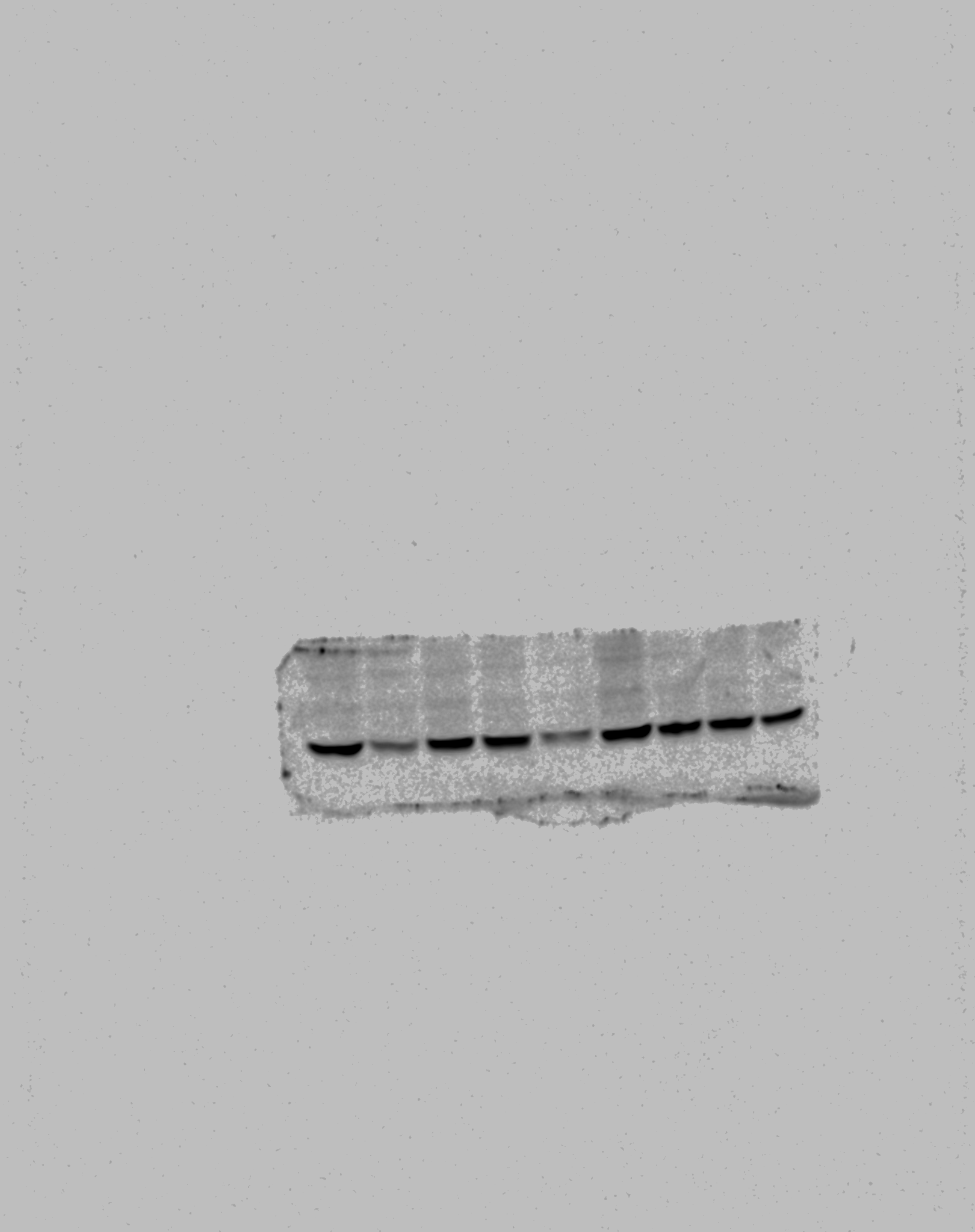

Supplement: Supplementary file 1 [file DataSheet_1.zip › supplementary materials-20190610/Figure5A-bcl2.bmp]

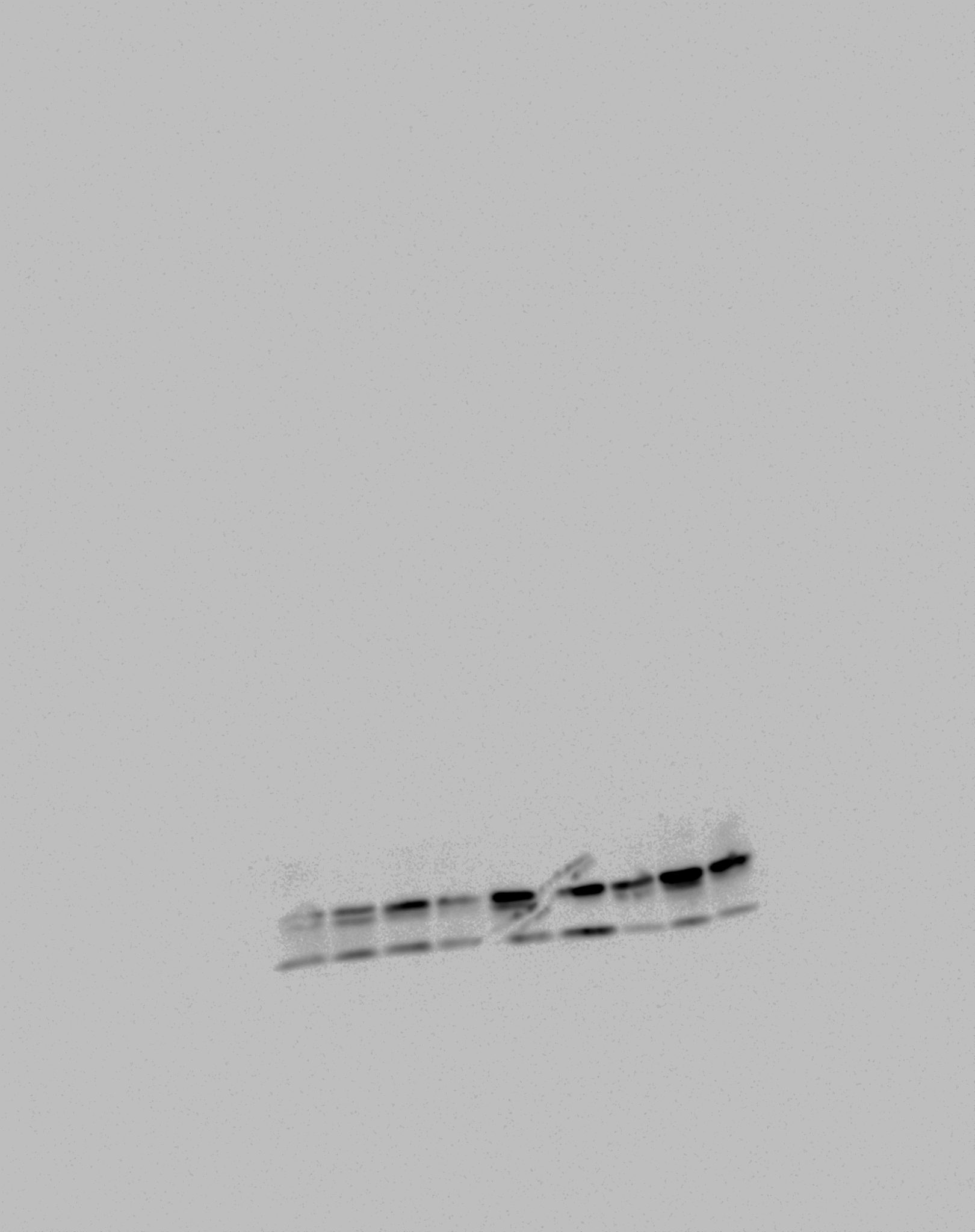

Supplement: Supplementary file 1 [file DataSheet_1.zip › supplementary materials-20190610/Figure5A-Caspase3.bmp]

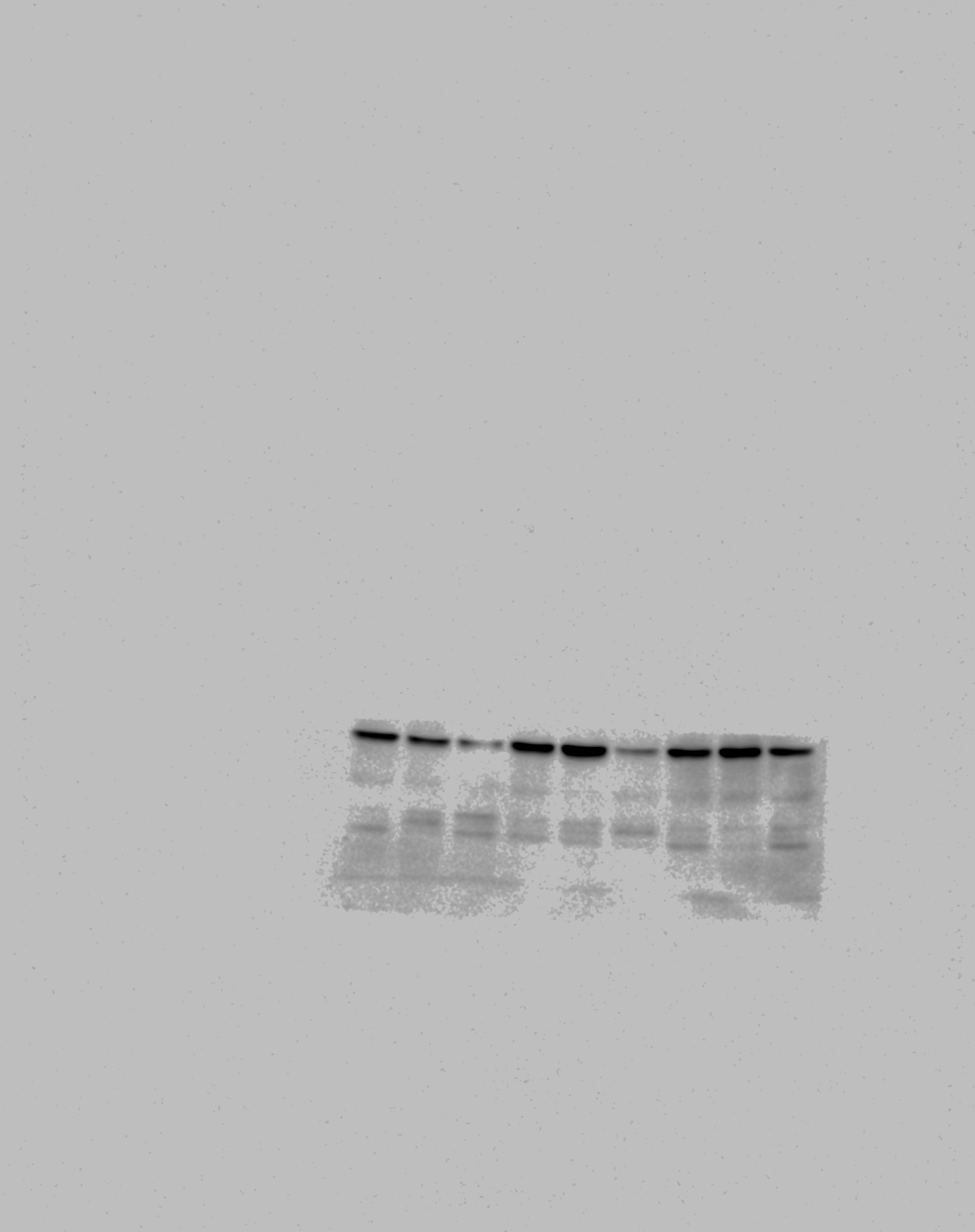

Supplement: Supplementary file 1 [file DataSheet_1.zip › supplementary materials-20190610/Figure5A-Cleaved-Caspase8-2.bmp]

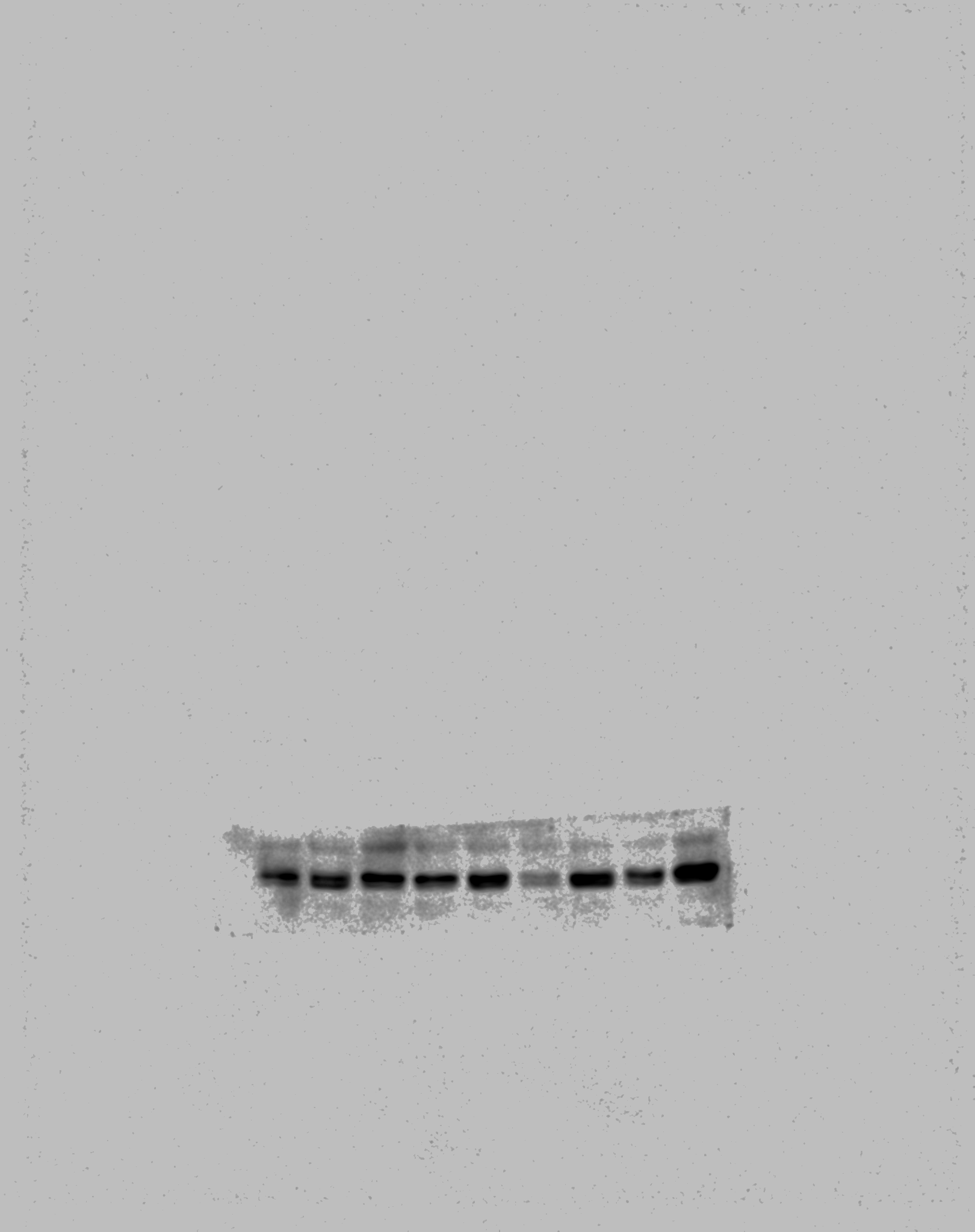

Supplement: Supplementary file 1 [file DataSheet_1.zip › supplementary materials-20190610/Figure5A-Cleaved-caspase9-2.bmp]

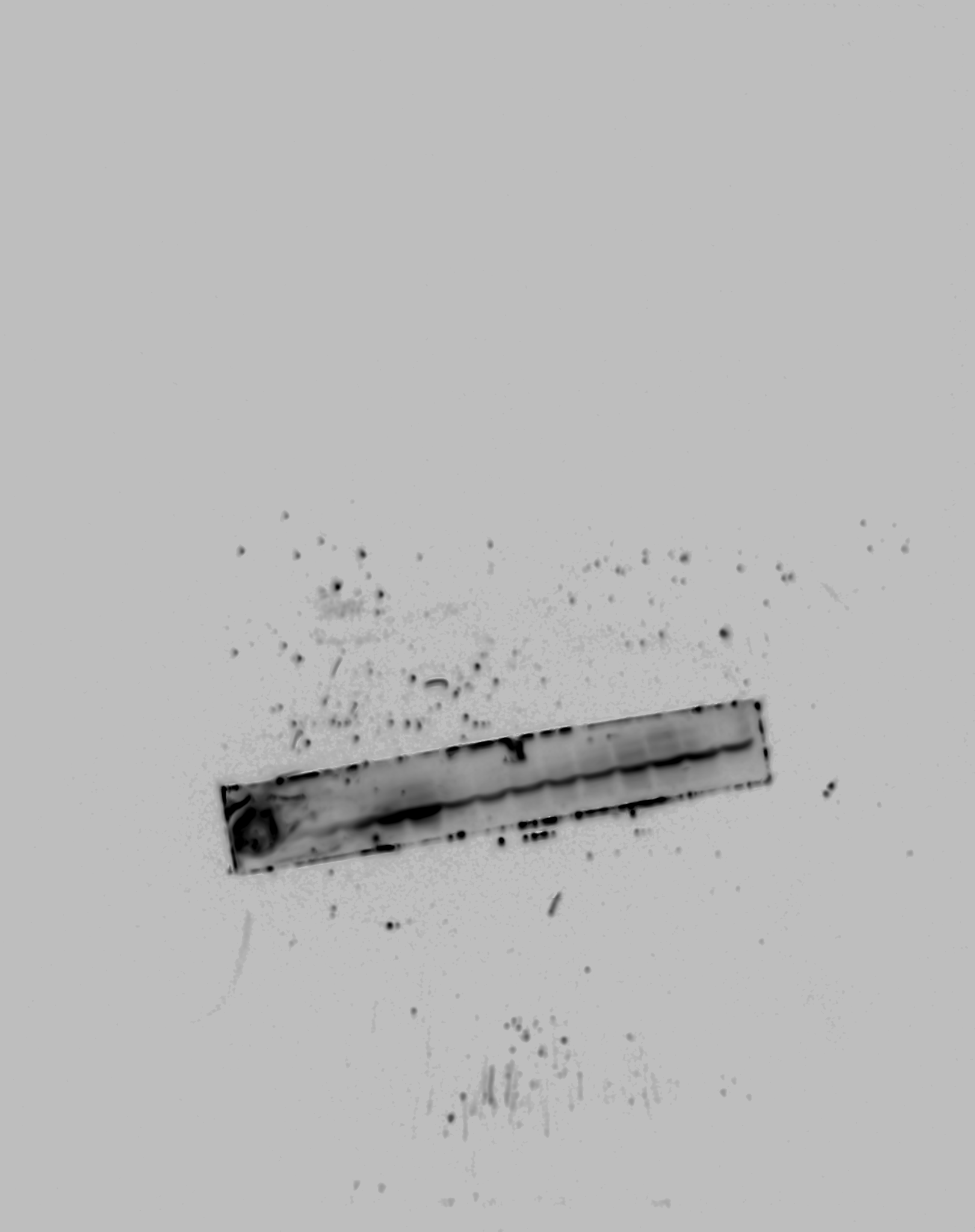

Supplement: Supplementary file 1 [file DataSheet_1.zip › supplementary materials-20190610/Figure5A-β-actin(last 9 lanes).bmp]

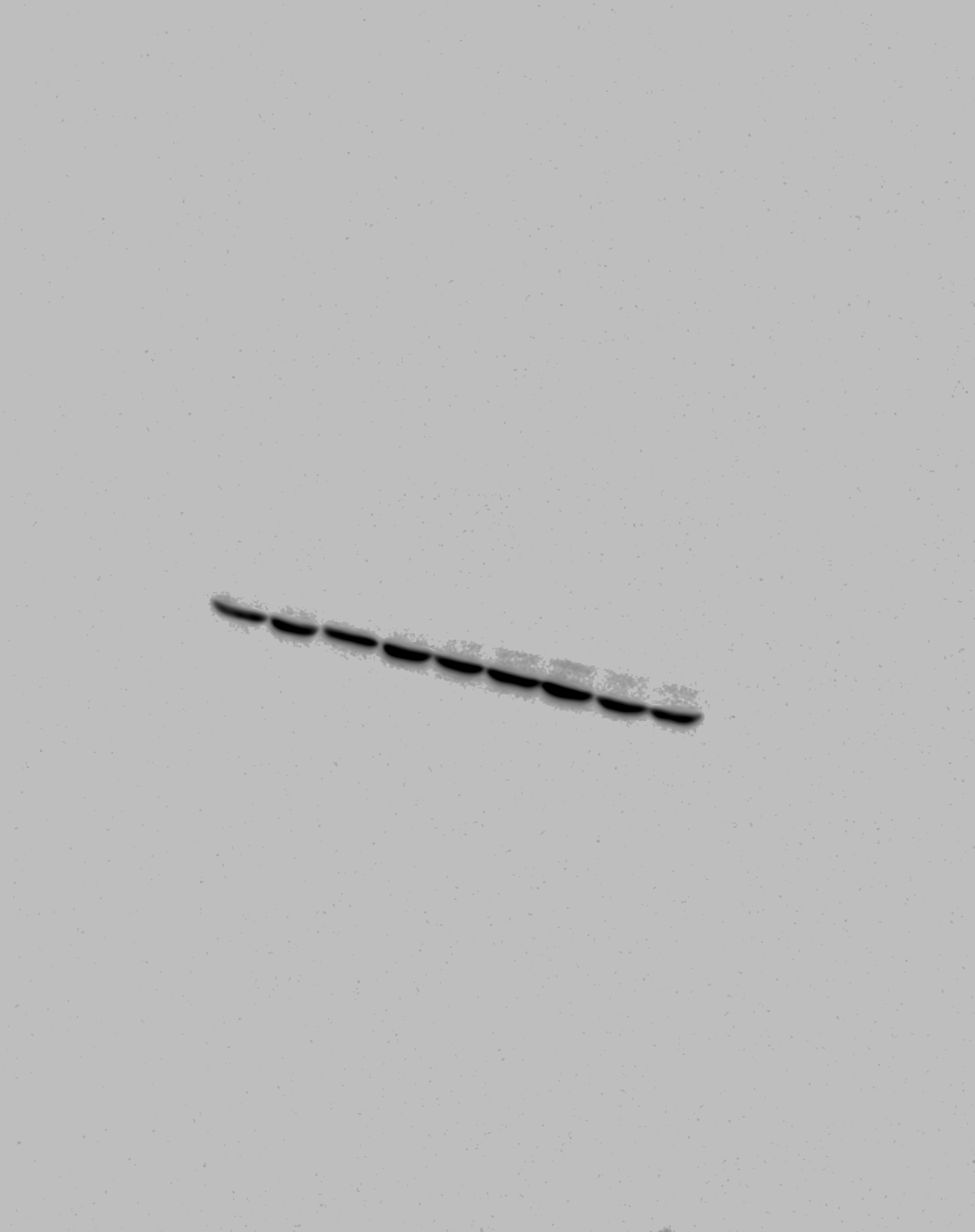

Supplement: Supplementary file 1 [file DataSheet_1.zip › supplementary materials-20190610/Figure6C-GAPDH.bmp]

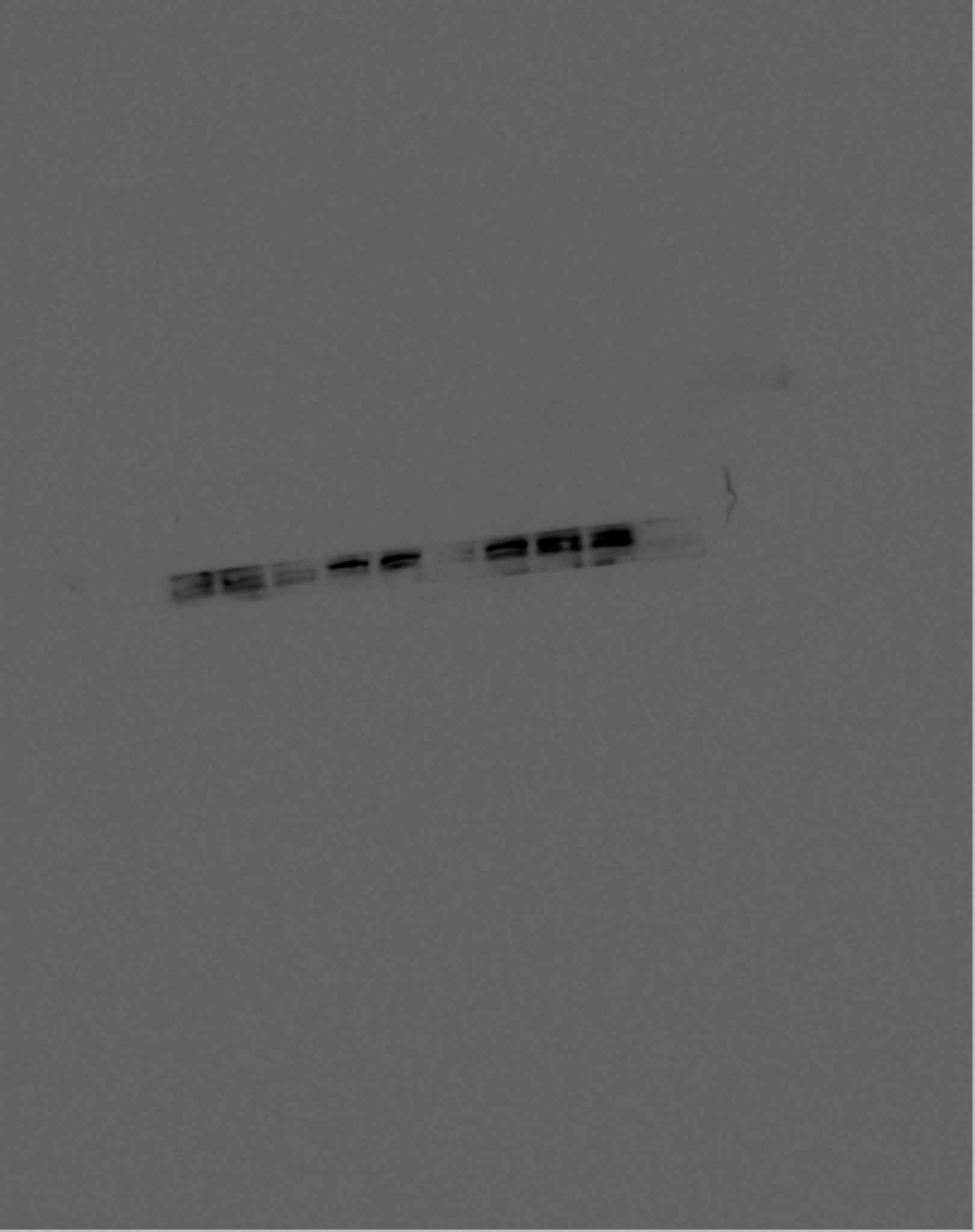

Supplement: Supplementary file 1 [file DataSheet_1.zip › supplementary materials-20190610/Figure6C-p-SMAD2.bmp]

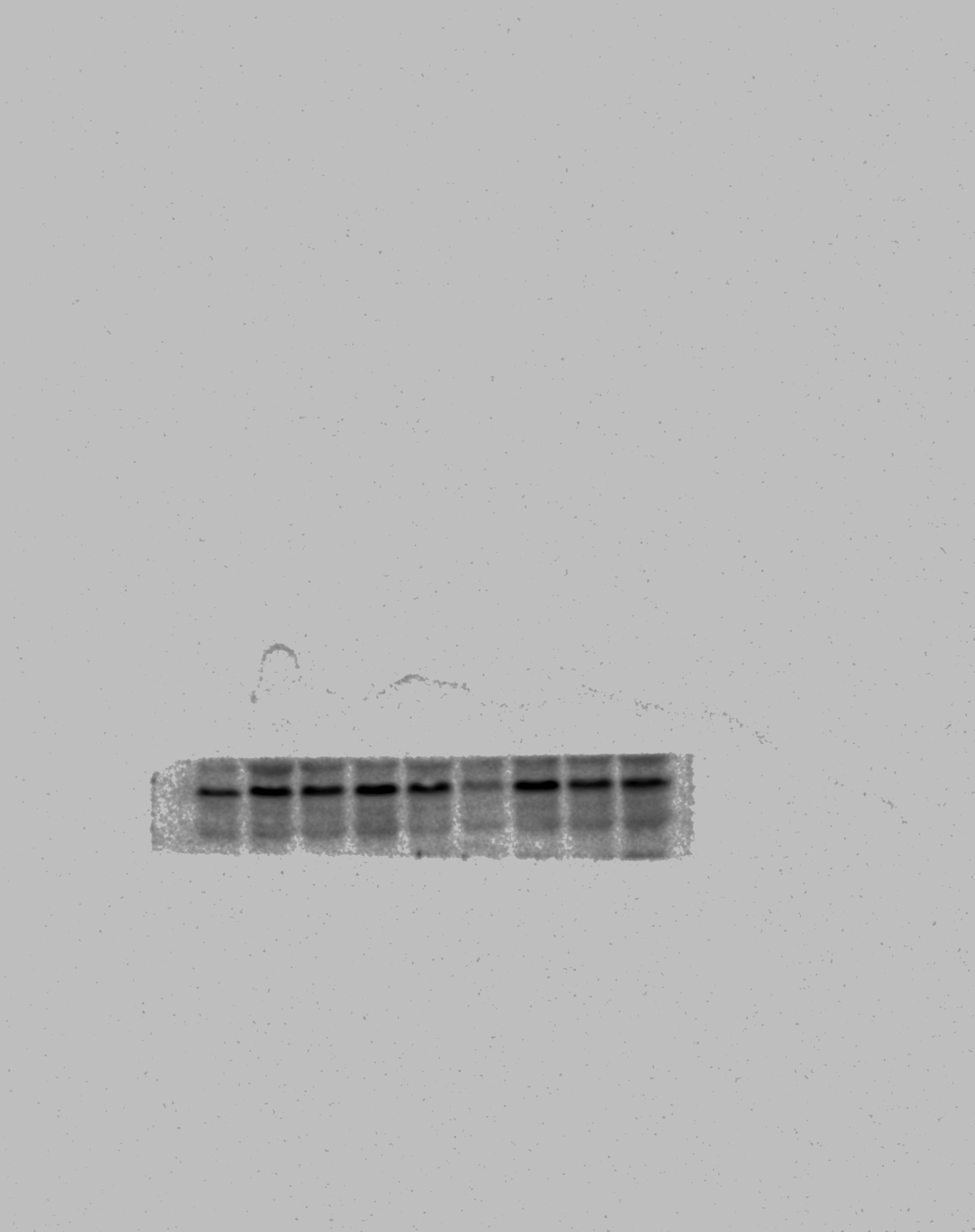

Supplement: Supplementary file 1 [file DataSheet_1.zip › supplementary materials-20190610/Figure6C-p-smad3.bmp]
